# Anti-biofilm, drug delivery and cytotoxicity properties of dendrimers

**DOI:** 10.5599/admet.1917

**Published:** 2024-02-14

**Authors:** Christian K. Ezeh, Marie E. U. Dibua

**Affiliations:** University of Nigeria, Department of Microbiology, Nsukka, Enugu State, Nigeria

**Keywords:** Dendrimers, biofilm, nanotechnology, quorum sensing, drug delivery, anti-biofilm activity

## Abstract

**Background and purpose:**

Treatments using antimicrobial agents have faced many difficulties as a result of biofilm formation by pathogenic microorganisms. The biofilm matrix formed by these microorganisms prevents antimicrobial agents from penetrating the interior where they can exact their activity effectively. Additionally, extracellular polymeric molecules associated with biofilm surfaces can absorb antimicrobial compounds, lowering their bioavailability. This problem has resulted in the quest for alternative treatment protocols, and the development of nanomaterials and devices through nanotechnology has recently been on the rise.

**Research approach:**

The literature on dendrimers was searched for in databases such as Google Scholar, PubMed, and ScienceDirect.

**Key results:**

As a nanomaterial, dendrimers have found useful applications as a drug delivery vehicle for antimicrobial agents against biofilm-mediated infections to circumvent these defense mechanisms. The distinctive properties of dendrimers, such as multi-valency, biocompatibility, high water solubility, non-immunogenicity, and biofilm matrix-/cell membrane fusogenicity (ability to merge with intracellular membrane or other proteins), significantly increase the efficacy of antimicrobial agents and reduce the likelihood of recurring infections.

**Conclusion:**

This review outlines the current state of dendrimer carriers for biofilm treatments, provides examples of their real-world uses, and examines potential drawbacks.

## Introduction

Infections caused by bacteria remain one of the major global health problems. Antibiotics have been used for bacterial infection treatments. However, selective pressure, mutation, inappropriate use of antibiotics, and inadequate diagnosis have resulted in the rapid emergence and proliferation of multi-drug-resistant bacterial strains [1]. Multidrug-resistant (MDR) bacteria are a worldwide public health problem that increases morbidity and mortality among infected people and has a detrimental influence on a variety of clinical outcomes, including those of patients receiving cancer treatment, transplant surgery, or intensive care unit care [2]. MDR bacteria such as methicillin-resistant *Staphylococcus aureus* (MRSA), vancomycin-resistant *Enterococcus* (VRE), multi-drug-resistant *Mycobacterium tuberculosis* (MDR-TE), and carbapenem-resistant *Enterobacteriaceae* (CRE) are considered serious public health problem by WHO which calls for immediate action to mitigate their threats [3]. A form in which bacteria develop resistance to antibiotics and other environmental stress is through the formation of biofilm. Bacterial biofilms are organized communities of bacterial cells attached and embedded in a self-formed extracellular polysaccharide matrix (EPS) [4]. The assembled microorganisms present in biofilms may consist of different or the same microbial species and typically grow on organic or inorganic surfaces that serve as a source of nutrients [5]. Biofilm formation by microorganisms confers advantages such as protection from adverse environmental conditions (desiccation and starvation), host immune defense systems, and antimicrobial substances [6].

These advantages make biofilm a serious public health problem, as seen in the difficulties in treating biofilm-associated infections. The health implication of biofilm can be seen in the data presented by the Center for disease control in 2007, which state that “in the USA, about 1.8 million nosocomial infections were biofilm-associated, leading to a severe economic loss of over $11 billion” [6]. Furthermore, biofilm-related infections are responsible for more than 500 deaths annually, with a high treatment cost of over $94 billion [7]. The majority of persistent infections in humans, such as chronic sinusitis, chronic otitis media, cystic fibrosis, valve endocarditis, and implant devices (urinary catheters, prosthetic joints, and heart valves), are biofilm-related [8,9].

Thus, treatment of biofilm-related infection is problematic due to the possession of structural dynamic properties such as EPS that limit diffusion of antimicrobial agents, rendering them inactive [1]. Biofilm eradication strategies involve the development of biofilm-inhibitory agents that prevent biofilm formation during the early stage and biofilm dispersal agents that interfere with biofilm cell assemblage. Antibiotics have been used to treat biofilms. However, biofilms are highly resistant to antibiotics [10]. Some anti-biofilm agents, such as antimicrobial peptides, quaternary ammonium compounds, antimicrobial lipids, anticancer drugs (mitomycin), and nitric oxide-releasing antibiotics, have been used for biofilm eradication. However, various disadvantages such as inherent structures and complicated nature of antimicrobial peptides, inherent toxicity of quaternary ammonium compounds and anti-cancer drugs, difficulty in handling nitric oxide, the activity of phenazines/quinolines against only Gram-positive organisms, and likely hold of developing resistance to antimicrobial lipids due to their presence in diets hinder the use of these agents [11]. Consequently, it is imperative to seek a more effective biofilm dissolution therapy. Recent advances in the field of nanotechnology have resulted in the development of nanomaterials and devices that have found useful applications in medicine. Interestingly, dendrimers have shown to be an alternative to conventional therapeutic agents in biomedicine and could be used to combat infections caused by multi-drug resistant pathogens, including those producing biofilm [12]. Dendrimers possess mono-dispersed and well-defined structures widely studied and applied in various biomedical fields, such as drug delivery systems, magnetic resonance imaging contrast agents, antiviral, and antitumor and antibacterial agents [13]. The high interest in dendrimers is due to their special properties, such as multi-valency, mono-dispersity, biocompatibility, high water solubility, and chemical modularity for multi-functionalization [14,15]. Multi-valency provides dendrimers with many different functional groups capable of interacting negatively with bacterial cell membranes, disrupting membrane integrity [14,15]. In addition, the deformable and flexible branches of dendrimers assist in the simultaneous binding of ligands and multiple receptors on a cell surface, inducing a strong avidity binding via the multivalent binding effect [16]. The application of dendrimers in controlling infections caused by microorganisms and biofilm eradication has been studied with more emphasis on its versatility as an antimicrobial agent and its structural ability to penetrate biofilm matrix. This review highlights biofilm formation, dendrimer classifications, functionalities, and its application as an anti-biofilm agent. Also, the limitations of dendrimers as drug delivery systems and anti-biofilm agents are summarized. Toxicity and strategies to modify dendrimers to overcome the various challenges in their application in conventional antimicrobial treatments are highlighted.

## Biofilm formation

**Figure 1.**
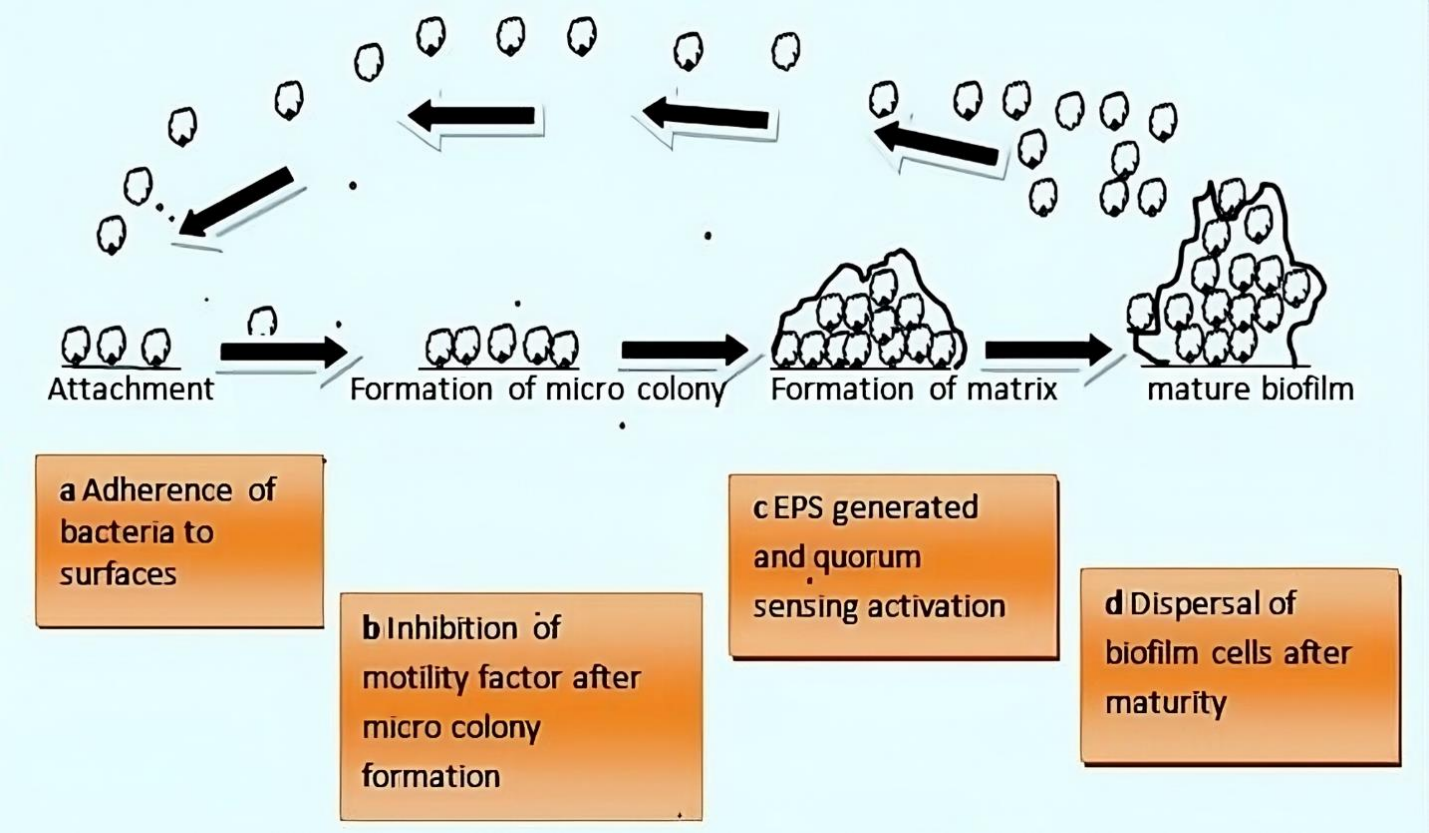
Biofilm formation

Bacteria exhibit two survival states: planktonic and biofilm (sessile). Bacteria form biofilms through complex and irreversible steps involving chemical, physical, and biological processes (Figure 1). Biofilm formation involves a series of stages. First, single planktonic cells roam and adhere to a surface. The surface provides a good conditioning environment for cell adherence for biofilm formation initiation. These cells become enclosed in exopolymeric materials. Adherent cells secrete an extracellular polymeric substance (EPS) and become irreversibly attached to the surface, resulting in microbial cell aggregation and matrix formation. The biofilm begins to grow and mature by forming water channels, microcolonies and water channel systems while also becoming extensively layered. When the biofilm matures, after reaching maximum cell density, it is considered a three-dimensional community. Lastly, the mature biofilm releases microcolonies of cells from the main community, allowing them to move freely to new surfaces and disseminate the infection to new areas [11].

### Attachment

The formation of a conditioning layer is the first stage in biofilm development. Fluid components settle onto the surface at this stage, thus forming a layer called substratum. Generally, rough surfaces and hydrophobic materials are more conducive than smooth and hydrophilic surfaces during biofilm formation [17]. These surfaces become inhabited by microorganisms, which cause them to produce a surface charge [18], which aids in the attraction and adhesion of microbial cells of opposing charges. The presence of glycocalyx, pili, and fimbriae on the surface makes the organisms cling to the surface more securely [19]. The preliminary stage is reversible but can be irreversible if adhesion is greater than repulsion.

### Growth (formation of microcolonies)

The bacteria community starts to grow once the adhesion is strong enough to utilize the nutrients. At this point, biological processes control how other materials adhere to surfaces. This is the outcome of multiple genes that produce surface proteins like porins being expressed [20]. The polysaccharides used to create the EPS layer are transported through porins. The microbial cells interact as the biofilm develops through the secretion of autoinducer signals (AIs) [20].

About 100 billion bacterial cells can be contained in a well-developed biofilm per milliliter. Hence, microbial communication is crucial. The microbial cells are divided into a wide variety of communities, and each community is in charge of a specific task [21]. Another typical occurrence in a developing biofilm is the formation of tall, wrinkled structures, which exerts lateral pressure on cells by pushing the cells against each other. Dead cells in biofilms localize in the regions that encourage vertical bulging, which aids in relieving this pressure [22].

### Matrix formation (metabolism)

The changes in the biofilm's environment result in changes in metabolic activity. The metabolic activity is high in the early stage of biofilm formation and gradually declines as development progresses [23]. Complex diffusion channels are utilized as the cell population grows for the circulation of nutrients, oxygen, and other components essential for cell growth. These channels are used to carry metabolic wastes and debris as well. The metabolic activities of the cells change in response to changes in the biofilm's environment. The gene expression in glycolysis is tightly regulated by conditions such as shear stress [24]. The bacteria that form biofilms tend to uptake foreign DNA, which could ultimately produce exogenous proteins [25]. Additionally, it was observed that some fatty acid-producing genes in some microorganisms are downregulated as biofilm develops [26].

### Dispersion

Dispersion is the last stage, characterized by the shedding of biofilm, allowing the sessile cells to revert to their former motile forms. Lastly, biofilm spreads, colonizes, and establishes new locations using their inherent powers. Cell-cell communication is crucial for the pathogenicity and growth of biofilms. Quorum sensing is the communication primarily carried by autoinducers (AIs) (small diffusible molecules). AIs differ in various biofilms and depend on the type of microorganisms involved [27].

## Composition of bacterial biofilm

Biofilms are made up of many components, such as the extra polymeric matrix (EPS) (which is the primary component), bacterial cells, secreted water, proteins, debris, and nucleic acid. These components make the free movement of materials and other important nutrients within the biofilm possible [28,29]. Biofilm arrangement is made primarily of two components: a region of tightly packed microbial cells with no obvious pores and a water channel for effective nutrient and other substance transport [30]. The configuration of microbial cells within biofilm determines the many physiological and physical characteristics of the biofilm.

Persister cells are also present in biofilm, rendering the human immune system and antibiotics ineffective. These are a few microbial cells that are resistant to antibiotic concentration and would normally be able to wipe out the majority of the bacterial population. The existence of these cells was discovered while researching the effect of penicillin on the *streptococci* population [31]. In addition to persisters, the biofilm activates various stress-related genes and factors that alter the resistant microbes to change properties such as temperature, pH, nutrition, osmolarity, and cell density, which alters the features of resistant microbes [32]. When circulatory systems and biofilm water channels were compared, their functioning was quite similar to that of early multicellular organisms [33].

Hydrodynamics and nutrient availability are two environmental elements that have an impact on biofilm formation and persistence. The biofilm is polymorphic, and studies with various glucose concentrations have shown that it can alter its form in response to the nutrients available in the environment. When the concentration of glucose rises, microbes quickly proliferate, and the thickness of the biofilm increases and vice versa [34]. Some studies have shown that different hydrodynamic conditions can alter the structure of biofilms. Bacterial microcolonies grow round in laminar flow, while in turbulent flow, they spread in the direction downstream [35].

## Biofilms induce antibiotic resistance

Bacteria in biofilms are characteristically more tolerant to antimicrobial agents than planktonic cells of the same strain. While the antibiotic resistance mechanisms of planktonic bacteria are well known, those mechanisms (efflux pump, mutation, and antibiotic modifying enzymes) are not the main antibiotic-resistant mechanisms of biofilms [36].

**Figure 2.**
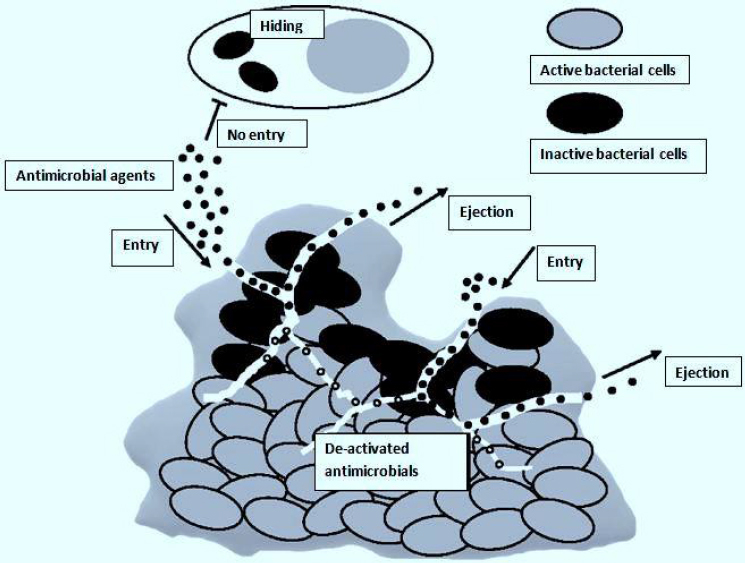
Fate of antimicrobial agents inside a biofilm matrix (ejection or de-activation). Adapted from Wang *et al.*[28] (copyright permission granted by Oxford Academic Publisher)

The biggest obstacle after entering the biofilm is breaking through the compact matrix of the biofilm [36]. The drug's availability inside the biofilm is greatly decreased because the EPS absorbs it and prevents it from spreading throughout the matrix [37]. Additionally, EPS is characterized by tiny pores that can prevent bigger drug molecules from passing through [38]. Consequently, the antimicrobial treatment needed to get rid of biofilms maybe 1000 times more than needed for planktonic cells [39]. Also, inside a biofilm, antimicrobial agents that have penetrated may be enzymatically inactivated [35]. Inadequate oxygen and nutrient levels result in microbial cells being buried in the polymeric matrix, which makes them enter a stationary growth phase [40]. This phase reduces their susceptibility to antimicrobials that depend on the active growth of microbes [41]. In general, these factors make it extremely challenging to eliminate microbial cells deeply embedded in biofilms, resulting in recurring infections despite therapy (Figure 2) [42].

The effects of antimicrobials, once built up inside a biofilm, primarily depend on breaking down cell walls and possibly interfering with intracellular metabolic processes [42]. However, these won’t happen due to the effective defense mechanisms developed by biofilms, thus shortening the presence of antimicrobial agents [43,44]. Microorganisms develop resistance to many antimicrobial agents through horizontal antimicrobial resistance gene transfer, spontaneous mutation, activity of efflux pumps, and acquisition of exogenous resistance genes (Figure 2) [40,45]. Microorganisms such as *Escherichia coli, Salmonella Typhimurium, Pseudomonas aeruginosa*, and *Candida albicans* are only a few of the species whose ejection of antimicrobial agents from their cells is linked with the over-expression of efflux pumps [46-48]. Additionally, sub-inhibitory antimicrobial exposure level may lead to cell-wall thickening of bacteria due to mutation, acting as a barrier restricting antimicrobial agent penetration and their uptake by the microbial cells [49]. This was observed in erythromycin's decreased antibacterial activity against methicillin-resistant *Staphylococcus aureus* (MRSA) [50].

Due to changes in transcription and proliferation rates, biofilm cells have a considerably increased frequency of horizontal gene transfer, resulting in various phenotypes [51]. Furthermore, the distinctive structure of biofilms creates nutrients, ionic strength, pH, redox potential across the biofilm, oxygen generation gradient, a corresponding variation in metabolic activity, and biofilm growth rate at various strata [52].

## Development of drug delivery systems

The eradication of infectious biofilms is a very difficult task. The use of traditional antimicrobial agents is complicated by numerous problems. To reach the target sites, antimicrobial agents must first "find their way" to a target without hurting the host. Naked antimicrobial agents without a drug delivery system frequently require a high dose to exert intended activities. This is because they lack target specificity and can harm the biological system [53]. Also, circulating antimicrobial agents can be deactivated by the immune system [54].

To activate a "stealth mode" and avoid the immuno-radar, an antimicrobial agent requires a pharmacological vehicle. Antimicrobial agents may not always be effective against resistant biofilm cells in laboratory experiments. If an adequate dose is used, increased antimicrobial resistance does not automatically indicate failure. The aforementioned difficulties would, however, be greatly exacerbated in a clinical setting because of a more complicated environment and the involvement of the host immune system [40]. To overcome these challenges, drug delivery methods that are target-oriented, biocompatible, non-immunogenic, and capable of penetrating biofilms and cell membranes must be created.

Potential prospects for eliminating pathogenic biofilms include developing a variety of site-specific targeting and effective penetrative drug delivery systems [55]. While antimicrobial resistance and recurrence of infections, as well as low pH settings, are shared characteristics of biofilms and tumors, they are also quite different from one other [56]. For instance, both of these have seen extensive applications of nanotechnology. Excellent biocompatibility, stability, and functionalization make nano-antimicrobial delivery systems target-oriented and environmentally sensitive [57].

In addition to the conventional chemical conjugation of antimicrobial agents to a carrier [58], a more appropriate method is to encapsulate antimicrobial agents into a vehicle that minimizes toxicity and unwanted side effects while protecting the cargo from degradation and deactivation [59]. These vehicles are also made to be exceptionally stable for parenteral injection, enabling the slow release of drugs at infection sites [60].

## Dendrimers as vehicles of antimicrobial agents targeting infectious biofilms

**Figure 3.**
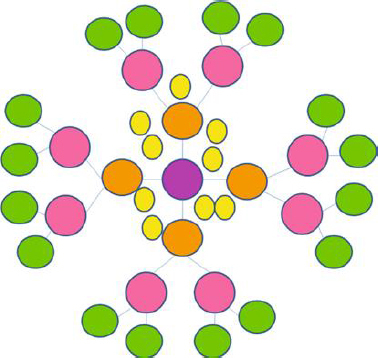
Basic structure of dendrimer. Adapted from Mittal *et al.* [61] (copyright permission granted by Hindawi)

Dendrimers are spherical, nanoscale particles that resemble a tree because their branches extend outward from a central point (Figure 3).

The pharmacokinetic profile of this macromolecule is greatly influenced by its structure, which is composed of a central core, repeating and highly branching subunits coupled to the former, and terminals attached with adjustable functional groups [62]. Its generation type (either half generation in the form of G0.5, G1.5, or full generation G1, G2, G3, G4, G5) is determined by the rise in branching in the form of radially homocentric layers, also known as dendrons, which is also indirectly responsible for the growth of its globular size. The loading capacity of dendrimers depends on generation, structure, and the peripheral conjugated functional group.

The capacity to encapsulate drugs concurrently rises with increased branching and internal core size [63]. Encapsulation, which entails enclosing the drug in the core hollow cavity, is one of the methods used for achieving drug delivery using dendrimers. Furthermore, covalent bonding with the surrounding functional groups can load antimicrobial agents onto the surface [13,64]. These techniques have long been used to synthesize various classes of dendrimers such as poly(amidoamine) (PAMAM), poly ether-copolyester (PEPE), liquid crystalline dendrimers, core-shell tecto dendrimer, poly(propylene imine) (PPI) dendrimers, glycodendrimers, polyglycerol, dimethylolpropionic acid dendrimers, chiral dendrimers, polyamido amine organosilicon dendrimers (PAMAMOS), poly(2,2-bis (hydroxymethyl) propionic acid dendrimers, and poly-L-lysine, poly(etherhydroxylamine) [62,65-71].

With its central core acting as the trunk and the branched dendrons arising from it, a dendrimer appears like a tree with many branches. The leaves of this branching, represented by the lateral functional groupings, are where it ends. The drug molecule is encapsulated by noncovalent interactions as a result of the interior cavity's click-in mechanism, which holds the drug moiety within the dendrimer and the number of branches surrounding the core cavity's shielding property from the outside environment.

When these weak hydrophobic and ionic bonds reach the optimal pH level, the amide group undergoes protonation, the host moiety separates, and the drug is released to the targeted cell [72]. Additionally, the charged functional groups on the surface of dendrimers exhibit effective electrostatic interaction with drug molecules of opposite charges. This kind of bonding improves the solubility of the drug. Drugs are conjugated with dendrimers via covalent bonds using specific chemical agents like polyethylene glycol (PEG), citric acid, macromolecules like saccharide [73], and para-aminobenzoic acid (PABA), which promotes increased stability, decreased toxicity, and controlled drug delivery [74,75].

Engineered dendrimers have a special ability for drug loading and delivery, and conjugating them with carbohydrates is fascinating for creating precise drug delivery systems. In addition to targeted delivery, dendrimers can be conjugated with carbohydrates to gain useful properties like bio-adhesion, stealth properties, solubility, biocompatibility, and decreased toxicity [63,76,77]. The therapeutic efficiency of antibiotics can be increased and adverse effects can be minimized by encapsulating them in dendrimeric systems. Controlling particle size, surface characteristics, functionality, branch length/density, and drug release are the main goals in the design of dendrimers as delivery systems to achieve the desired impact at the designated site of action [78]. The active molecules may condense inside the dendrimers, adhere to their surface chemically or physically, or both. These structures enhance the pharmacokinetic and pharmacodynamic characteristics of pharmaceuticals and can be used with more conventional drugs [79].

The PAMAM dendrimer is one of the most studied dendrimers for the release of antibacterial agents because of its hydrophilic characteristics, which are derived from many surface functional groups, making conjugation with antibacterial agents simple. The antibacterial capabilities can be improved when these dendrimers interact with water-soluble antibiotics. By substituting PEG or lauroyl chains for the amino-terminal groups of PAMAM dendrimers, it is possible to increase the substance's biocompatibility. Fluoroquinolones (nadifloxacin and prulifloxacin) antibacterial activity and water solubility significantly increased when conjugated with PAMAM G4 dendrimers containing ethylene-diamine surface groups (64 NH2 groups) [80,81]. To evaluate the resistance of *Staphylococcus aureus* and *Cryptococcus pneumoniae* strains to it, ciprofloxacin was loaded on basic PPI and PEGylated PPI dendritic structures. The antibacterial activity of the dendrimer loaded with ciprofloxacin was much higher than that of each of the individual components, showing that the conjugated system works in tandem [82].

## Dendrimers

Dendrimers are globular hyperbranched nanopolymeric molecules with homogeneous, distinct, and monodisperse structures. They are made up of a central core moiety, repeating branching chains, peripheral reactive functional groups, and size ranges between 1 to 10 nm [83]. Dendrimers are classified based on their monomers, and their examples include polyamidoamine (PAMAM), polypropyleneimine (PPI), arborols, chiral dendrimers, and liquid crystalline, core-shell (tecto) [84].

The chemical and physiological properties of dendrimers depend on their monomers, generations (size), terminal groups, and synthetic routes. These parameters can be modified by dendrimers with specific properties for application in different biomedical fields [85]. While their characteristic properties vary, they have numerous characteristics that make them distinctive from other drug carriers. Explicitly, their biocompatibility, high degree of branching, water solubility and polyvalency make dendrimers an ideal carrier for various antimicrobial agents [86]. The numerous terminal functional groups of dendrimers, for instance, aid the conjugation of a wide range of biologically active moieties, such as chemotherapeutic agents, targeting agents, and nucleic acids. Dendrimers can also make it easier for numerous therapeutic agents to bind to their individual target molecules simultaneously by combining them into a single nanostructure [87]. This boosts the therapeutic efficacy.

## Synthesis of dendrimers

Dendrimers are synthesized using two main methods: divergent and convergent methods. In the divergent method, the synthesis starts from the central core outward and presents (branches) functional groups for attachment or substitution with monomers. This method obtained the first generation (G1). This step is followed by the removal of inactive monomers, allowing for further binding of more monomers to ensure dendrimer growth [88]. In a study by Tomalia *et al.* [89], PAMAM dendrimer was synthesized using ammonia as the starting core; it underwent three Michael addition reactions with methyl acrylate. The terminal ester groups reacted with more ethylenediamine, forming a G1 dendrimer. Further amidation and Micheal additions resulted in newer generations [88].

Meanwhile, in the convergent method, synthesis starts from the monomers outside to the inside core [88]. The basic structure of the output molecule is predetermined and calculated by counting the branches connected to it. In this type of synthesis process, the new periphery molecule is activated for various reactions with monomers [90]. The convergent method is more advantageous because it has better structural control due to the low probability of side reactions, reduced amount of reagents, and production of pure compounds due to the purification process involved with each step [88]. However, the production of higher-generation dendrimers using the convergent method is challenging due to the steric effect observed in these macromolecules [91].

## Classification of dendrimers

### Based on property

Despite having a similar geometric architecture, dendrimers differ in terms of their physical and chemical characteristics.

**Hydrophilic dendrimers:** Generally, the most widely synthesized and commercialized are PAMAM dendrimers. Michael addition reaction is the first reaction that occurs in between an alkyl diamine core utilizing monomers of methyl acrylate to form a branched intermediate. The newly formed monomers, the reaction between ethanolamine and excess ethylenediamine, can be transformed into two smaller generation molecules, such as OH or -NH group surface group moieties, respectively [92].

This intermediate releases the smallest anionic dendrimers possessing four COOH groups on hydrolysis of the methyl group. When dendrimer development exceeds a certain threshold, the synthetic yield declines. The decline is due to the steric effect as a result of congestion of the branching arms (dense parking effect). Additionally, because of their increased water solubility, distinctive structure, and extensive range of surface groups, they are considered appropriate vehicles for the delivery of antimicrobial agents. They are available as methanol solutions and are obtainable commercially. The subclass of dendrimers with a tris-aminoethylene-imine core is known by the commercial name Starburst® dendrimers [92].

**Biodegradable dendrimers:** To create an ideal and huge molecular weight polymer with a high tissue deposition and quick clearance through urine to prevent nonspecific toxicity, biodegradable dendrimers were developed. In physiological solutions, they are frequently created by adding ester groups via enzymatic or chemical cleavage. The determining elements are the size of dendrimers, the monomeric unit’s lipophilicity, the type of chemical bonds, and the cleavage susceptibility of the internal and external dendrimer structures. Due to their biocompatibility and biodegradability, polyester dendrimers are used in gene therapy and anticancer treatments. However, the current study has switched to finding specific spatial and temporal degradation characteristics rather than the non-specific hydrolysis process and long-term deterioration characteristics [93].

**Figure 4.**
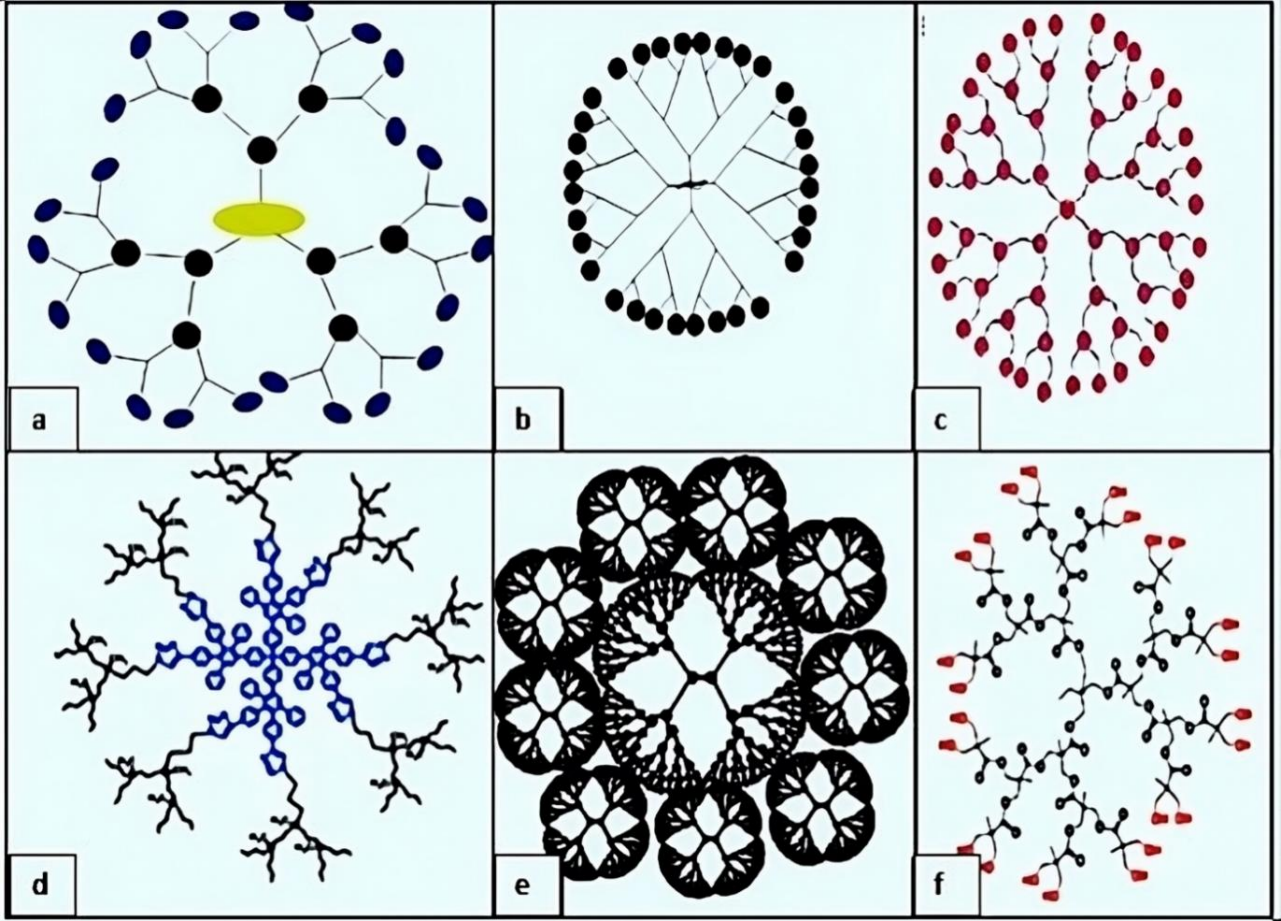
Some types of dendrimers and their structures (a) peptide dendrimers, (b) glycodendrimer, (c) chiral dendrimer, (d) hybrid dendrimer, (f) polyester dendrimer. Adopted from Alfei&Schito [12] (copyright was granted by MDPI publishers)

**Amino acid-based dendrimers**: Blocks with various features, including hydrophobicity, optical property, chirality, and biorecognition, were integrated to create amino acid (AA) dendrimers. Generally, chirality in an atom is usually created by the joint action of the core and branching unit molecules with surface terminating groups. The unique internal structure created by amino acid building blocks offers stereoselective locations for non-covalently attaching molecules. Dendrimers are employed as targeted drug delivery systems, gene carriers, and protein mimics due to their distinctive structural folding of the unique branching units. These families of dendrimers are often created by grafting amino acids (AAs) or peptides into a regular dendrimer surface or by attaching AAs or peptides to an organic or peptide core (Figure 4a) [93].

**Glycodendrimers:**The interaction of carbohydrates with numerous receptors on the cell surface, which in turn regulates several normal and aberrant processes, provides the basis for creating glycodendrimers. It was discovered that this interaction was potent for a multivalent ligand-receptor system. The findings from numerous studies led to the conclusion that carbohydrates were utilized as carriers in dendrimers. According to reports, glycodendrimers are used as a metastatic agent, an immune stimulant, and a carrier for cancer treatment (Figure 4b) [93].

**Hydrophobic dendrimers:** Water solubility must be high enough for dendrimers to be delivered systemically. However, the dendritic structure's hydrophobic void regions support the improved encapsulation and solubilization of lipophilic components. This structure resembles the micelle of an amphiphilic polymer, but it lacks the critical micellar concentration (CMC). Dendrimer building blocks are covalently bonded to one another, preventing them from disintegrating during the diluted solution phase. The solubility of hydrophobic dyes, probes, and fluorescent markers has been explored successfully in dendrimers, which have been found to exhibit hydrophobic interior gaps and hydrophilic surfaces mimicking unimolecular micelles.

Cyclophanes or dendrophanes are dendrimers reported to contain aliphatic and aromatic components. It has also been reported that these forms of dendritic structure regulate the release of drugs [93].

**Asymmetric dendrimers**: The bow tie polyester dendrimers (Figure 4f), which Gillies & Fréchet [94] formulated, are the most well-known asymmetrical dendrimers and may have a better pharmacokinetic profile. These are typically created by joining dendrons from different generations to a core linear molecule, forming a non-uniform orthogonal dendritic structure. In this form of dendrimer, the molecular weight, structure, and quantity of functional groups can be adjusted. Lee *et al.* [95] created a G3 asymmetric dendrimer via click chemistry.

### Based on structure

**Simple dendrimers:** Simple monomeric units make up these kinds of dendrimers, which result from symmetrical substitution of benzene tricarboxylic acid ester. They contain 45 molecular diameters and 4, 10, 22, and 46 benzene rings connected symmetrically [96].

**Crystalline dendrimers:** Mesogenic monomers are used to form this kind of dendrimers by functionalizing carbosylane [97].

**Chiral dendrimers**: The chirality in these sorts of dendrimers depends on the construction of four constitutionally distinct but chemically related branches to a chiral core, such as chiral dendrimers made from pentaerythritol (Figure 4c) [98].

**Micellar dendrimers:** These dendrimers are water-soluble, completely aromatic, and hyperbranched polypropylene dendrimers that can produce a cluster of aromatic polymeric chains that can resemble some micellar structures and form complexes with tiny organic molecules in water [98].

**Hybrid dendrimers:** Peripheral amines in zero-generation polyethyleneimine undergo functionalization modifications, which results in the formation of these dendrimers. Columnar and cubic-like structures with a variety of structural characteristics arise as a result, and these structures experienced significant modification to give rise to dendritic structures such as hybrid dendritic linear polymers (Figure 4d) [99].

**Amphiphilic dendrimers:** They are prepared by segregation in which two sides of a chain are frequently split, with one side having electron-withdrawing capabilities and the other having electron-donating properties. These substances include, for instance, superfecta, hydraamphiphiles, and bolaamphiphiles [99].

**Metallodendrimers:** Metallodendrimers are formed using a complex method that can occur either inside the molecule or on its periphery. These dendrimers, such as ruthenium bipyridine, were discovered to have electrochemical and luminescent characteristics [96].

**Tectodendrimers:** Commercial tectodendrimers include Starburst® and Stratus® CS Acute Care^TM^. They have dendrimers in the center, and their functions range from identifying sick cells to determining the presence of infections (Figure 4e) [96].

**Multilingual dendrimers:** A multilingual dendrimer that is commercially available is VivaGel. On the surface, it contains several copies of a specific group of functions [98].

**Multiple antigen peptide dendrimers**: The poylysineskelton is used to generate a dendron-like structure in multiple antigen peptide (MAP) dendrimers. The amino acid lysine aids in conjugating the side chain of the alkylamine, which serves as a monomer for the different branching units. These dendrimers were formed and discovered to have a wide range of biological applications in diagnosis and vaccine production [100].

## Drug encapsulation in dendrimers

The phenomena of drug release by dendrimers are dependent on the type of dendrimer and core moieties used. Different processes, including electrostatic encapsulation, covalent conjugation, and physical encapsulation, are used in drug release patterns [100].

### Physical mode of encapsulation

By altering their shapes, cavities, and structural layouts, the molecules are entrapped in the inner moiety of the macromolecule using this technique. The internal cavities remain vacant, having two groups, lipophilic and hydrophobic interactions, which cause an interaction with the medicament molecules of nitrogen or oxygen atoms along with the release of the hydrogen bond. Several interactions, including physical and hydrogen bonding, led to the hydrogen bonding. Several drugs, including anticancer drugs like doxorubicin hydrochloride and methotrexate, can be encapsulated using this method [101].

### Electrostatic interactions

Because dendrimers have several NH_2_ and COOH groups employed to increase the solubility of lipophilic drugs, the interaction in this type of encapsulation occurs on their surface. Easily ionizable drugs like ibuprofen, ketoprofen, diflunisal, naproxen, and indomethacin form complexes with multifunctional surfaces of dendrimers having terminal groups [101].

### Covalent conjugation

This conjugation technique is employed when the compound has functional groups on its surfaces. In this technique, hydrophilic labile linkages are broken down chemically and enzymatically to form conjugated molecules. In addition, the stability and kinetics of the drug can be improved by using a spacer, as is the case with penicillin V, 5-aminosalicylic acid, venlafaxine, propranolol, and naproxen conjugated with PAMAM dendrimers. Other spacers include polyethylene glycol, aminobenzoic acid, lauryl chains, and p-amino hippuric acid. The consequence is increased solubility and regulated release of medications [102].

## Applications of dendrimers in biofilm control

**Figure 5.**
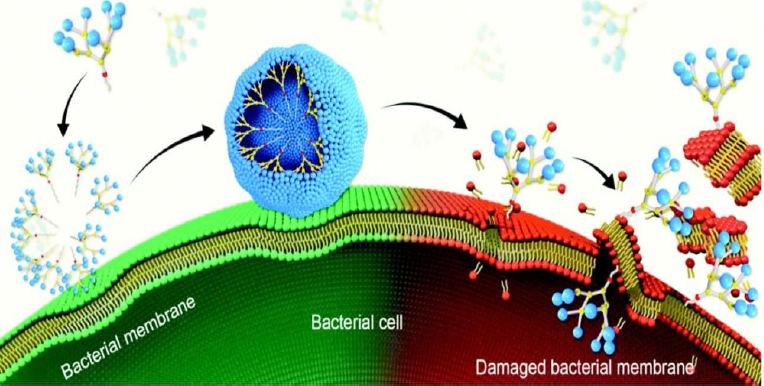
antibacterialactivity illustration of amphiphilicdendrimers throughmembraneadsorption,self-assembling,interaction,insertion,disintegrationanddisruption. Adapted from Dhumal *et al.* [106] (copyright was granted by Royal Society of Chemistry publishers)

Highly branched three-dimensional structures called dendrimers have cavities that can be filled with both hydrophilic and hydrophobic substances [103]. Low-weight dendrimers have been reported to exhibit antimicrobial activity against *S. aureus* and *E. coli* without any loaded antibiotics [104] (Figure 5). Antibiofilm activities have been investigated in various studies. It was reported that the fucose-peptide dendrimer was highly effective in preventing *P. aeruginosa* biofilm formation by disrupting their membrane attachment [105].

In another investigation, the antibiofilm activity of metallodendrimers comprising ruthenium(II) or copper(II) complexed with carbosilane dendrimer was evaluated. These metallodendrimers were found to inhibit *S. Aureus* biofilm formation and low hemolysis activity was observed [107]. Similarly, Han *et al.* [108] investigated the antibiofilm activity of lapidated TNS18 peptide dendrimer against *P. aeruginosa* biofilm. It was reported that TNS18 peptide dendrimer inhibited 90 % of biofilm formation, utilizing half of its minimum inhibitory concentration (MIC). *P. aeruginosa's* swarming motility was distorted at the same concentration by the dendrimer, and 55 % of the biofilms were dispersed at a 16-fold MIC concentration. Furthermore, Gide *et al.* [109] investigated the effectiveness of lipidated AMP-based dendrimers in preventing biofilm development. The dendrimer showed antibacterial action against both planktonic and biofilm cells and gram-negative and gram-positive bacteria, including MDR strains. By creating lysine branches, the researchers optimized the length of the fatty acid chain. The most effective substance, D-A-2, had a minimal hemolytic effect while being able to damage the membranes of MRSA and *E. coli*. Additionally, D-A-2 was able to completely halt the growth of *E. coli* and MRSA biofilms at a dosage of 0.8 μg/mL.

In another study, researchers investigated the role that various auxiliary groups played in dendrimer penetration of *P. aeruginosa* biofilms. Dendrimers with NH_3_^+^ groups at their perimeter penetrate *P. aeruginosa* biofilms' acidic environment more quickly than dendrimers with OH or COO groups. Electrostatic forces also led the peripherally charged dendrimers with NH_3_^+^ groups to be drawn to the negatively charged components of the biofilm, leading to aggregation around the top of the biofilm. Dendrimers possessing peripheral groups (OH and COO^-^) accumulate more frequently and are evenly distributed when they penetrate biofilms than NH_3_^+^ dendrimers. This means that the surface makeup of dendrimers can precisely control how well they penetrate and accumulate in biofilms, which is a crucial discovery for the ongoing development of novel antibacterial or antimicrobial-carrying polymers [110].

A study investigated the anti-biofilm activity of polyamidoamine dendrimers and polyaminophenolic ligands against mono- and multi-species legionella biofilms formed by *L. pneumophila* in conjunction with other bacteria prevalent in tap water, such as *Escherichia coli, Aeromonas hydrophila, Klebsiella pneumonia* and *Pseudomonas aeruginosa*. Cytotoxicity assay was carried out to test the concentrations of chemicals used as antibiofilm agents. The highest non-cytotoxic chemical concentration was used for biofilm inhibition activity, with dendrimer concentration tenfold being 10 times greater than polyaminophenolic ligands. Among the polyaminophenolic ligands chemicals, macrophen and double macrophen were the most active. Dendrimers were twofold more effective when compared with other chemicals, with a reduction of up to 73 and 85 % of multi-species biofilm and Legionella, respectively. These findings imply that the investigated compounds, particularly dendrimers, could be considered novel molecules in planning studies to create effective anti-biofilm disinfection methods for water systems to reduce legionellosis outbreaks [111].

The galactose-specific lectin LecA partly mediates the formation of antibiotic-resistant biofilms by *P. aeruginosa*, an opportunistic pathogen causing severe respiratory infections in cystic fibrosis and immunocompromised patients, suggesting that preventing LecA binding to natural saccharides might provide new opportunities for treatmentIn a study, convergent chloroacetyl thioether (ClAc) ligation between digalactosylated dendritic arms and 8-fold or 4-fold chloroacetylated dendrimer cores resulted in the formation of *P. Aeruginosa* biofilm inhibitor and 8-fold (G3) and 16-fold (G4) galactosylated analogs of GalAG2, a tetravalent G2 glycopeptide dendrimer LecA ligand. Biofilm inhibition assays, hemagglutination inhibition, calorimetry, and isothermal titration revealed that G3 dendrimers bind LecA slightly more effectively than their parent G2 dendrimers and cause complete biofilm inhibition and *P. aeruginosa* biofilm dispersal, whereas G4 dendrimers exhibit reduced binding and no biofilm inhibition. Based on the crystal structure of G3 dendrimer LecA complex, a binding model is formed to explain the observed saturation of glycopeptide dendrimer galactosyl groups and LecA binding sites [112].

A contributing factor in the development of antibiotic resistance in the opportunistic bacteria *Pseudomonas aeruginosa* is the establishment of biofilm, which prevents drug penetration. Hence, research focused on modifying tetravalent glycopeptide dendrimer ligands of *P. aeruginosa* lectins LecA or LecB to increase their antibiofilm activity. First, heteroglycoclusters were examined, showing one pair each of LecA-specific galacosyl groups and LecB-specific fucosyl groups and then binding simultaneously to both lectins, one of which provided the first fully resolved crystal structure of a peptide dendrimer as LecB complex, providing a structural model for dendrimer-lectin interactions (PDB 5D2A). By adding more cationic residues to these dendrimers, biofilm inhibition was increased, but bactericidal effects were equivalent to those of non-glycosylated polycationic antimicrobial peptide dendrimers. Another strategy involves creating dendrimers with four copies of Lewis^a^ (a natural LecB ligand), which resulted in biofilm inhibition and slightly stronger LecB binding. Finally, excellent biofilm inhibition and dispersal were achieved by combining the antibiotic tobramycin with a LecB-specific nonbactericidal antibiofilm dendrimer at sub-inhibitory concentrations of both substances [113].

To probe if LecB inhibition affects these processes, high-affinity ligands were obtained by screening two 15,536-member combinatorial libraries of multivalent fucosyl-peptide dendrimers. The most effective LecB ligands observed were dendrimers PA8 (OFuc-LysAlaAsp)4(LysSerGlyAla)2 LysHisIleNH2 (IC50 = 0.11 mM by ELLA) and FD2 (C-Fuc-LysProLeu)4(LysPheLysIle)2 LysHisIleNH2 (IC50 = 0.14 mM by ELLA). Dendrimer FD2 activity resulted in the complete inhibition of *P. aeruginosa* biofilm development (IC50 10mM) and also led to the complete dispersal of already established biofilms in several clinical and wild-type strains of *P. aeruginosa* isolates. These studies imply that LecB inhibition by highly-affinity multivalent ligands can be a therapeutic strategy for treating *P. aeruginosa* infections by preventing the development of new biofilms and the spread of existing ones [114].

G3KL and TNS18 peptide dendrimers prevent the growth of *Pseudomonas aeruginosa* biofilms below their MIC value by affecting their swarming motility, as reported by Han *et al.* [107]. A higher concentration above the MIC was, however, needed to eradicate the preformed biofilm. Following observation using a scanning electron microscope and confocal laser micrographs, peptide dendrimers were shown to destroy biofilm morphological structure completely in a dose-dependent fashion. Long hydrophobic alkyl chains with tiny hydrophilic poly(amidoamine) dendrons with different terminal functionalities make up amphiphilic dendrimers. Astonishingly, the amphiphilic dendrimer containing amine terminals demonstrated strong antibacterial action against both Gram-positive and Gram-negative bacteria as well as drug-resistant bacteria, and it inhibited the formation of biofilms [106].

## Advantages and limitations of dendrimers as a drug carrier

Dendrimers are hyperbranched polymers created in layers around a central core [115]. These structures are, therefore, very reactive to bacteria in vivo due to their larger surface area to size ratio and hydrophilic structure. Additionally, these structures possess a high density of functional groups attached for enhanced targeted delivery [115].

The properties of dendrimers, including chemical reactivity, solubility, and glass transition temperature, depend on the nature of the end group. The solubility of dendrimers varies with the diversity of functional groups. When hydrophilic groups are present, they are highly soluble in polar solvents, and when hydrophobic groups are present, they are soluble in nonpolar solvents [116]. Their higher generation results in an increase in cubic volume. Dendrimers have a great degree of structural control and are typically compact, spherical, or globular in shape. They have high ionic conductivity, reactivity, water, and non-polar solubility.

Dendrimers are a great choice for target bio-imaging and diagnostics because of their ability to adjust their properties based on their shape, solubility, mono-dispersity, and the simplicity with which huge doses of substances can be loaded [117]. To improve the selectivity and bioavailability of hydrophobic drugs at the point where they bind to biofilms, several approaches are being used. Also, clearance through the reticuloendothelial system is considerably reduced due to its size [62]. Dendrimers feature surface functional groups that can act as vectors to target a particular spot and deliver drugs specifically to biofilms.

**Figure 6.**
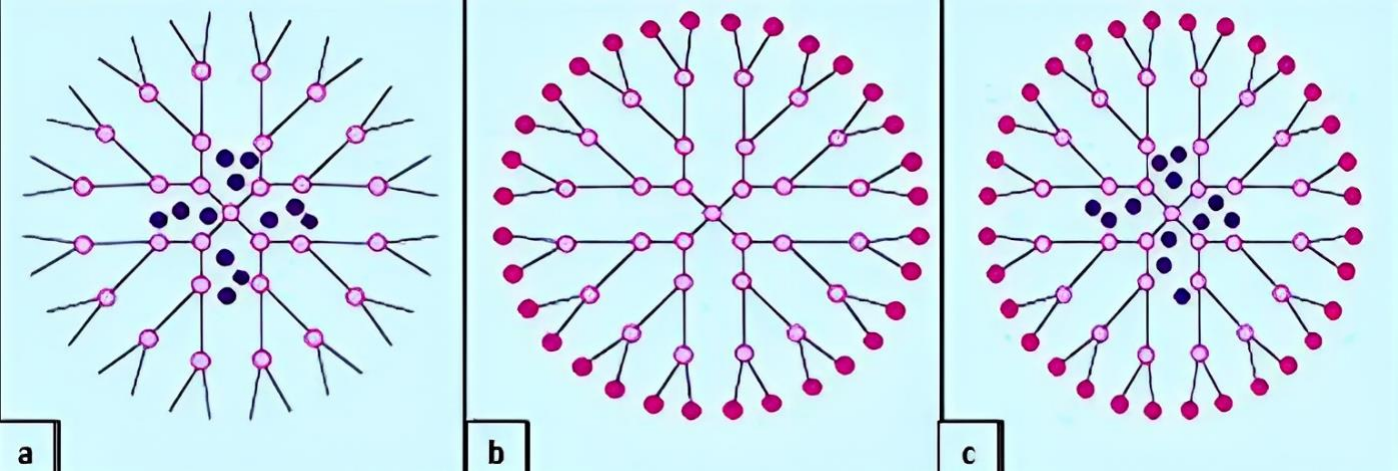
A basic representative pattern of conjugating molecules in dendrimers: (**a**) internal cavities encapsulation, (**b**) peripheral attachment, (**c**) peripheral attachment and internal cavities encapsulation simultaneously. Adapted from Janaszewska *et al.* [119] (copyright permission granted by MDPI publishers)

Due to their structural specificity, dendrimers make ideal partners for active pharmaceutical ingredients, which allows the following: inclusion of pharmaceutical compounds inside the cavities (Figure 6a), attachment of pharmaceutical compounds to the functional groups at the periphery of the dendrimer (Figure 6b), and both offering encapsulation (internal cavities) and support for conjugates (on the surface) (Figure 6c).

The interaction between pharmaceuticals and dendrimers is advantageous because it increases solubility, which enhances the drug's toxicity, absorption, and bioavailability [118].

Dendrimers provide several advantages over other polymeric architectural systems. Dendrimers' branched macromolecule properties are entirely distinct from modern linear polymeric materials [83]. Such variations are frequently caused by the method of synthesis. While the methods for developing dendrimers can produce homogeneous structures with uniform molecular weights, conventional polymerization typically results in polydisperse structures with various molecular weights [120]. Dendrimers are referred to as "host-guest" molecules because of their spherical shape and internal chambers, which allow them to demonstrate excellent encapsulation properties and convey many substances in their interior. Two or more molecules/ions are held together in host-guest systems by unique structural connections. They are interconnected by forces other than fully covalent bonds [121].

The high solubility and permeability of dendrimers have reportedly been attributed to the presence of many surface functional groups. The terminal groups can be very reactive at times, necessitating additional changes. It is possible to change a macromolecule's physicochemical properties or create a specific function, such as a catalytic or medicinal one, by post-modifying its surface [122].

The dendritic architecture and several secondary amine groups in the structure of the dendrimers provide more drug-loading reaction sites. Engineered dendrimers have a special ability for drug loading and delivery, and conjugating them with carbohydrates is fascinating for creating precise drug delivery systems. In addition to targeted delivery, dendrimers can be conjugated with carbohydrates to gain useful properties like bioadhesion, stealth properties, solubility, biocompatibility, and decreased toxicity [77].

Despite these unique features, their usage is restricted because of their rapid in vivo clearance and significant cytotoxicity under physiological settings [123,124]. Regardless of dendrimers' advantages as drug delivery vehicles, there are still certain problems to be resolved. Dendrimer toxicity and biodistribution are tightly correlated with their size and surface chemistry [122]. The main issue is a size restriction. Generation 6 and higher PAMAM dendrimers require a greater reliance on the hepatic clearance pathway for clearance, while generation 5 or lower dendrimers can effectively be removed via glomerular filtration and the renal excretion pathway [125]. Dendrimers with diameters ranging from 4 to 10 nm can cross the cellular endocytosis barrier and interact with nanometric cellular components [122]. However, generation 6 and higher PAMAM dendrimers are quite expensive and poisonous [64]. As a result, PAMAM dendrimers from higher generations are rarely employed. Cationic dendrimers have a strong ability to bind to nuclei or anion molecules, which help to internalize cells [62]. However, non-specific plasma protein adsorptions and reticuloendothelial system-accelerated elimination are frequent problems for cationic dendrimers [122]. Additionally, there is a limited intracellular dissociation of dendrimers with nucleic acids [126]. Cationic dendrimers are more hazardous than neutral or anionic dendrimers, especially at high dosages, since their interaction with cell membranes (negatively charged) can promote cell membrane instability, leading to cell lysis [127,128].

In research where dendrimers were tested in already-existing biofilms, disruption was not seen despite the encouraging results against planktonic bacteria. The researchers speculated that the dendrimers' huge size, which prevents their entry into the biofilm's matrix and limits them to the surface of the biofilm, may be the cause of their ineffectiveness against mature biofilms [129]. Additionally, the dendrimers demonstrated toxicity in cytotoxic tests utilizing the A549 human lung cancer cell line and hemolysis assays at concentrations within the same order of magnitude as the MIC values determined for the examined bacterial strains [129]. According to the authors, this data shows that dendrimers have a wide range of activities on both bacterial and human cells. These dendrimers have antibacterial action, but their antibiofilm activity needs to be enhanced with reduced cytotoxicity for therapeutic applications. Therefore, the efficiency of dendrimers is influenced by the length of the alkyl chain, dendrimer synthesis, and bacterial strain [130,131].

## Cytotoxicity of dendrimers

As a potential therapeutic agent, dendrimers have shown significant contributions in the biomedical field. However, just like any other therapeutic agent, toxicity study is of great importance to assess its safety for biological applications. As highlighted earlier, the toxicity of these nano-carriers is related to their size and structure. Dendrimers are nanosized, enabling them to interact with many other cellular components, including nucleic acids, proteins, plasma membranes, ions, heavy metals, vitamins, and organelles (mitochondria, endosomes, nuclei) [62].

In general, the toxicity of dendrimers can be related to their physicochemical properties, such as size, generation, charge, and concentration. The cytotoxicity of dendrimers is dependent on size and generation. The cytotoxicity of low-cationic generation is normally lower than that of high-generation dendrimers due to the increased number of positive charges associated with them [132]. It was reported by Han *et al.* [133] that PAMAM G3 caused 80 % hemolysis after 24 h, G4-NH2, and G5-NH2 dendrimers caused 100 % hemolysis within 4 h, while PAMAM G6-NH2 dendrimers call caused total hemolysis in 2h.

It has been shown that the toxicity of dendrimers is highly dependent on surface charge (anion, cation, and neutral). Cationic dendrimers exhibit a high level of cytotoxicity when compared to anionic dendrimers [134]. An *in vivo* study using an embryonic zebrafish model showed that surface greatly influences the toxicity of dendrimers [128]. Positively charged dendrimer cores showed considerable cytotoxicity and drastic phenotypic alterations when dendrimers were used as nanocarriers, in contrast to negatively charged dendrimer cores [135]. The maximum tolerance value (MTV) can be used to express the toxic concentrations of cations; a value above the MTV demonstrates concentration-dependent toxicity, whereas anions and neutrals hardly exhibit cytotoxicity over a wide range of concentrations (except for extremely high concentrations), both *in vivo* and *in vitro*. In an *in vivo* test, the MTV cytotoxicity of cationic dendrimers (PAMAM) in the mouse stomach ranged from 30-200 mg/kg, while the MTV of anionic dendrimers reached as high as 500 mg/kg (below G7) [136]. Naturally, hemolytic concentration is another logical indication of toxicity evaluation in addition to MTV. High-production cation dendrimers demonstrate the highest levels of hemolytic toxicity and cytotoxicity (at 90 nM, G6). High-generation cations have substantial toxicity at low concentrations. However, they do not exhibit toxicity below a particular level (below 0.009 nM, G6) [133].

There are different ways of evaluating the toxicity of therapeutic agents, either by assessing their activity against microbial cells, body cells, or tissues. This review focused more on the cytotoxicity of dendrimers on the body cells and tissues. By staying in circulation for extended periods and staying in contact with blood flow components, dendrimers enhance sustained systemic distribution [137]. However, when cationic dendrimers enter the blood, they interact with the proteins and cells (RBCs, white blood cells, and platelets) to cause hemolytic toxicity and alter hematological parameters.

Cationic PAMAM dendrimers G4, G5, and G6 were demonstrated to cause the aggregation of human platelets, as measured by particle size and surface charge analyses, rather than by altering the integrity of the plasma membranes of platelets [138]. Dendrimer nanocarriers prevent immunotoxicity but can also have the opposite effect on the body [139]. By using immunoprecipitation and Ouchterlony double-diffusion experiments, Roberts *et al.* [140] attempted to determine the immunogenicity of cationic PAMAM dendrimers G3, G5, and G7 with an amino surface in rabbits. The blood-brain barrier (BBB) prevented the majority of drug delivery methods from accessing the brain, making drug delivery in the central nervous system difficult. A significant issue is how harmful drug delivery systems are to the central nervous system [141]. Dendrimers can carry drugs or genes to treat brain or nervous system diseases over the BBB, but it is important to consider their potential for neurotoxicity [142]. Similar to dendrimers, dendrimers' structural features have been shown to cause a variety of neurotoxicological effects in living things. Following oral administration, PAMAM dendrimers can be physiologically distributed in the heart, lungs, liver, blood, urine, stomach, and small and large intestine [143]. Tissue accumulation of Organs like potential reactive oxygen species (ROS) producing dendrimers, wide distribution of phagocytes, and particularly, the slow clearance of dendrimers renders organs like the spleen and liver the main target of dendrimers toxicity. In addition, dendrimer exposure may impact high-blood flow organs, including the kidneys and lungs [144]. Few researchers, however, have looked at the organ-specific toxicity of dendrimers. More thorough toxicity studies on each organ are required for the safety of administration [145]. Specific toxicity assay of organs will be vital to control the dose and reduce potential side effects. The presence of autophagic vesicles in hepatocytes, hepatocyte necrosis, and vacuolation was seen in mouse liver sections after exposure to 100 g/mL of PAMAM dendrimer G5 for 24 h. When mice were given dendrimers, the administration of the autophagy inhibitors 3-methyladenine (3-MA) and chloroquine (CQ) led to the recovery of liver weight loss, a reduction in liver tissue damage, and the blocking of serum biochemical parameters, which suggests that PAMAM dendrimers may damage liver tissue through autophagy [146]. Dendrimers can penetrate the digestive tract after oral administration and cause harmful effects on the gastrointestinal epithelium [147]. In isolated rat jejunum displayed in chambers, PAMAM dendrimers were demonstrated to penetrate the intestinal epithelium. However, 1 mM G3.5 and G4 PAMAM dendrimers failed to promote paracellular transport to isolated rat tissue and harmed rat jejunal cells. This discrepancy might have occurred because the supporting cells and mucus beneath the epithelial barrier of the rat jejunal mucosa were less susceptible to permeability than the Caco-2 cell culture [148].

Following local administration of PAMAMNH2 G2 and G3 dendrimers (at a dosage of 6 mg/mL), skin irritation tests on rats demonstrated minor erythema and evident alterations in the shape of epidermal cells. Rats treated with PAMAM-NH2 at much higher doses (30 or 300 mg/mL) exhibited moderate to severe erythema as well as clear histological alterations in the dermis. Additionally, when exposed to high doses of PAMAM-NH2 dendrimers, PCNA was strongly expressed in all skin layers and the nuclear immune response was boosted, suggesting cell proliferation disorder. Immunohistochemical studies and microscopic evaluations revealed that cationic PAMAM dendrimers quickly caused significant skin toxicity and that low concentrations should be considered for real-world external application [149].

## Dendrimers modification

Dendrimers can be used as the foundation for bigger nanoparticles through the development of certain strategies. For instance, dendrimer clusters can create size-switchable or adaptable nanoparticles [149]. Most of them are 100 nm in size, and they don't move around while blood circulates. When nanoparticles reach the desired tissues, they break apart and precisely release individual dendrimers, allowing them to use their exceptional tissue penetration ability and cell internalization properties. Goa *et al.* [150] created charge and size adaptive clustered nanoparticles based on the electrostatic interaction between PAMAM and 2,3-dimethyl maleic anhydride modified poly(ethylene glycol)-block-polylysine (PEG-b-PLys) [150]. The outside layer of the clustered nanoparticle is made up of PEG chains, and the inner core is made up of complexes of PAMAM with PLy chains. The clustered nanoparticles were larger than 100 nm and contained peripheral PEG chains. They were shown to have prolonged blood circulation and a negatively charged surface of about -2.2 mV. The dendrimer and PEG-b-PLys were broken down when they reached the sick lung tissue because the acidic microenvironment caused the carboxyl groups in the PLy segments to change to amine groups. The released PAMAM dendrimers successfully penetrated and were retained inside biofilms by utilizing their tiny size (6.5 nm) and positive charge (23.8 mV). Charge-reversal dendrimers for surface stealth modification during body circulation, PEG is a non-immunogenic, inert, non-antigenic polymer with great biocompatibility and water solubility. It has been approved by FDA [151].

Dendrimers are frequently PEGylated to protect their cationic surfaces, lessen their toxicity, and extend their circulation times [152]. By lowering unspecific absorption, PEGylated PAMAM dendrimers lengthen systemic circulation duration and boost the concentration in the target tissues [153].

Deactivating the cationic surface charges of dendrimers is another method to lessen non-specific cellular uptake and adsorption during blood circulation. The positive surface charges can then be activated inside the target tissues or cells. Based on this idea, charge-reversal or charge-switchable dendrimers can be created. Parameters such as temperature, osmotic pressure, pH, and biological signals may be employed to reverse charges on the dendrimer surface [56,154].

Another method of reducing the toxicity of dendrimers is through acetylation. When the acetyl group is conjugated with the terminal group, the positive charges on the surface of the dendrimer are neutralized. It was observed that acetylated dendrimers exhibit higher solubility, which is a special property for drug development and biological applications [119]. In addition, attention needs to be given to anionic or half-generation dendrimers. This is because previous research has shown that the toxicity of dendrimers is caused by the presence of polycationic charges on their surface, and full-generation dendrimers are characterized by the presence of polycationic functional groups, while half-generation contains the carboxylic acid functional group. Hence, research should be focused on half-generation to improve its biocompatibility. Also, dendrimers with negative charges on their surface were shown to exhibit negligible toxicity and hemolytic effects [155].

## Conclusions

Bacterial pathogens have developed many mechanisms to resist antimicrobial agents, and the formation of biofilm remains one of their most effective resistance mechanisms. Biofilm formation by pathogens protects the organisms against antimicrobial agents and from adverse environmental conditions (desiccation and starvation) and the host immune defense systems. Hence, biofilm-associated microbial infection constitutes a serious public health problem. Recent advances in nanotechnology and the fabrication of various nanomaterials and dendrimers have shown enormous promise. Dendrimers showed a tremendous ability to inhibit biofilm formation and development due to their special properties such as -dispersity, biocompatibility, high water solubility, and chemical modularity for multi-functionalization. Also, due to their unique structural organization, drugs can be incorporated into dendrimers through molecular modifications. However, just like other antimicrobial and antibiofilm agents, dendrimers showed moderate cytotoxicity related to their size, generation, charge, and concentration. Despite dendrimers' advantages as drug delivery vehicles, there are still certain problems to be resolved. Dendrimer toxicity and biodistribution are tightly correlated with their size and surface chemistry. Modification of these parameters can help shape dendrimers to be effective antibiofilm and drug-delivery agents to curtail the menace of biofilm-related infections. Advancement in computational modeling and analytical techniques is necessary for future research because it will provide a deeper understanding of dendrimer properties and activity, accelerating the development of new dendrimers with improved features. This can be tailored to design potent and selective dendrimers that can effectively target and disrupt biofilms with less cytotoxicity.

## References

[1] Fan X. Yang F. Ma C.N. L. Cheng C. Haag R.. Biocatalytic nanomaterials: A new pathway for bacterial disinfection. Advance Materials 33 (2021) 2100637. https://doi.org/10.1002/adma.202100637 10.1002/adma.202100637 PMC11468881 34216401

[2] Antibiotic Resistance Threats in the United States, (Department of Health and Human Services, CDC, 2019). https://www.cdc.gov/drugresistance/pdf/threats-report/2019-ar-threats-report-508.pdf

[3] Serwecinska L.. Antimicrobials and antibiotic-resistant bacteria: A risk to the environmental andto public health. Water 12 (2020) 3313.https://doi.org/10.3390/w12123313 10.3390/w12123313

[4] Kannappan A. Gowrishankar S. Srinivasan R. Pandian S.K. Ravi A.V.. Antibiofilm activity of *Vetiveriazizanioides* root extract against methicillin-resistant *Staphylococcus aureus*. Microbial Pathogenesis 110 (2019) 313-324. https://doi.org/10.1016/j.micpath.2017.07.016 10.1016/j.micpath.2017.07.016 28710012

[5] Padmavathi A.R. Bakkiyaraj D. Pandian S.K.. Biofilm inhibition by natural products of marine origin and their environmental applications, in Biofilms in Plant and Soil Health, Ahmad I. Husain F. M., Eds., Wiley, Hoboken,, Belgium, 2017, p. 465. https://doi.org/10.1002/9781119246329.ch23 10.1002/9781119246329.ch23

[6] Banerjee D. Shivapriya P.M. Gautam P.K. Misra K. Sahoo A.K. Samanta S.K.. A review on basic biology of bacterial biofilm infections and their treatments by nanotechnology-based approaches. Proceedings of the National Academy of Sciences B 90 (2019) 243-259.https://doi.org/10.1007/s40011-018-01065-7 10.1007/s40011-018-01065-7

[7] Schrekker C.M. Sokolovicz Y.C. Raucci M.G. Selukar B.S. Klitzke J.S. Lopes W. Leal C.A.M. de Souza I.O.P. Galland G.B. Dos Santos J.H.Z. Mauler R.S. Kol M. Dagorne S. A L. Teixeria M.L. Morais J. Landers R. Fuentefria A.M. Schrekker S.. Multitask imidazolium salt additives for innovative poly (l-lactide) biomaterials: morphology control, Candida spp. biofilm inhibition, human mesenchymal stem cell biocompatibility, and skin tolerance. ACS Applied Material Interfaces 8 (2016) 21163-21176. https://doi.org/10.1021/acsami.6b06005 10.1021/acsami.6b06005 27486827

[8] Zhao Y. Dai X. Wei X. Yu Y. Chen X. Zhang X. Li C.. Near-infrared light-activated thermosensitive liposomes as efficient agents for photothermal and antibiotic synergistic therapy of bacterial biofilm. ACS Applied Material Interfaces 10 (2018) 14426-14437. https://doi.org/10.1021/acsami.8b01327 10.1021/acsami.8b01327 29651836

[9] Soleimani N. Mobarez A. Olia M. Atyabi F.. Synthesis, characterization and effect of the antibacterial activity of chitosan nanoparticles on vancomycin-resistant Enterococcus and other gram negative or gram positive bacteria. International Journal of Pure and Applied Sciences and Technology 26(26) (2015) 14-23. https://www.researchgate.net/publication/337472318_Synthesis_Characterization_and_Effect_of_the_Antibacterial_Activity_of_Chitosan_Nanoparticles_on_Vancomycin-_Resistant_Enterococcus_and_Other_Gram_Negative_or_Gram_Positive_Bacteria

[10] Mishra S. Gupta A. Upadhye V. Singh S.C. Sinha R.P. Häder D.P.. Therapeutic Strategies against biofilm infections. Life 13 (2023) 172. https://doi.org/10.3390/life13010172 10.3390/life13010172 36676121 PMC9866932

[11] Verderosa A.D. Totsika M. Fairfull-Smith K.E.. Bacterial Biofilm Eradication Agents. Frontiers in Chemistry 7 (2019) 824. https://doi.org/10.3389/fchem.2019.00824 10.3389/fchem.2019.00824 31850313 PMC6893625

[12] Alfei S. Schito A.M.. From Nanobiotechnology, Positively Charged Biomimetic Dendrimers as Novel Antibacterial Agents. Nanomaterials 10(2020) 2022.https://doi.org/10.3390/nano10102022 10.3390/nano10102022 33066468 PMC7602242

[13] Alfei S. Caviglia D.. Prevention and eradication of biofilm by dendrimers: A possibility still little explored. Pharmaceutics 14 (2022) 2016.https://doi.org/10.3390/pharmaceutics14102016 10.3390/pharmaceutics14102016 36297451 PMC9610720

[14] Palmerston Mendes L. Pan J. Torchilin V.P.. As nanocarriers for nucleic acid and drug delivery in cancer therapy. Molecules 22(22) (2017) 1401. https://doi.org/10.3390/molecules22091401 10.3390/molecules22091401 28832535 PMC5600151

[15] Teunissen A.J.P. Burnett M.E. Prévot G. Klein E.D. Bivona D. Mulder W.J.M.. Embracing nanomaterials’ interactions with the innate immune system. WIREs Nanomedicine and Nanobiotechnology 13(13)(2021) e1719. https://doi.org/10.1002/wnan.1719 10.1002/wnan.1719 33847441 PMC8511354

[16] Myung J.H. Gajjar K.A. Saric J. Eddington D.T. Hong S.. Dendrimer-mediated multivalent binding for the enhanced capture of tumor cells. AngewandteChemie International Edition 50(50) (2011) 11769-11772. https://doi.org/10.1002/anie.201105508 10.1002/anie.201105508 PMC3549433 22012872

[17] Wang Y. Lee S.M. Dykes G.. the physicochemical process of bacterial attachment to abiotic surfaces: challenges for mechanistic studies, predictability and the development of control strategies. Critical Review in Microbiology 41 (2015) 452-464. https://doi.org/10.3109/1040841X.2013.866072 10.3109/1040841X.2013.866072 24635643

[18] Yin W. Wang Y. Liu L. He J.. Biofilms: the microbial “protective clothing” in extreme environments. International Journal of Molecular Sciences 20(20) (2019) 3423. https://doi.org/10.3390/ijms20143423 10.3390/ijms20143423 31336824 PMC6679078

[19] Kostakioti M. Hadjifrangiskou M. Hultgren S.J.. Bacterial biofilms: development, dispersal, and therapeutic strategies in the dawn of the postantibiotic era. Cold Spring Harbor Perspectives and Medicine 3 (2023) a010306. https://doi.org/10.1101/cshperspect.a010306 10.1101/cshperspect.a010306 PMC3683961 23545571

[20] Cohen B.E.. Functional linkage between genes that regulate osmotic stress responses and multidrug resistance transporters: challenges and opportunities for antibiotic discovery. Antimicrobial Agents and Chemotherapy 58(58) (2014) 640-646. https://doi.org/10.1128/aac.02095-13 10.1128/aac.02095-13 24295980 PMC3910827

[21] Rasamiravaka T. Labtani Q. Duez P. El Jaziri M.. The formation of biofilms by Pseudomonas aeruginosa : a review of the natural and synthetic compounds interfering with control mechanisms. Biomedical Research International 2015 (2015) 759348 .https://doi.org/10.1155/2015/759348 10.1155/2015/759348 PMC4383298 25866808

[22] Asally M. Kittisopikul M. Rue P. Du Y. Hu Z. Cagatay T. Robinson A.B. Lu H. Garcia-Ojalvo J. Suel G.M.. Localized cell death focuses mechanical forces during 3D patterning in a biofilm. Proceedings of the National Academy of Sciences 109(109) (2012) 18891-18896. https://doi.org/10.1073/pnas.1212429109 10.1073/pnas.1212429109 PMC3503208 23012477

[23] Singh B.N. Prateeksha U.D.K. Singh B.R. Defoirdt T. Gupta V.K. Vahabi K.. Bactericidal, quorum quenching and anti-biofilm nanofactories: a new niche for nanotechnologists. Critical Review in Biotechnology 37 (2016) 525-540. https://doi.org/10.1080/07388551.2016.1199010 10.1080/07388551.2016.1199010 27684212

[24] Islam N. Kim Y. Ross J.M. Marten M.R.. Proteome analysis of Staphylococcus aureus biofilm cells grown under physiologically relevant fluid shear conditions. Proteome Sciences 12 (2014) 21. https://doi.org/10.1186/1477-5956-12-21 10.1186/1477-5956-12-21 PMC4013085 24855455

[25] Qayyum S. Sharma D. Bisht D. Khan A.U.. Protein translation machinery holds a key for transition of planktonic cells to biofilm state in Enterococcus faecalis: a proteomic approach. Biochemical and Biophysic Research Community 474 (2016) 652-659. https://doi.org/10.1016/j.bbrc.2016.04.145 10.1016/j.bbrc.2016.04.145 27144316

[26] Tielen P. Rosin N. Meyer A.K. Dohnt K. Haddad I. Ja¨nsch L. Klein J. Narten M. Pommerenke C. Scheer M. Schobert M. Schomburg D. Thielen B. Jahn D.. Regulatory and metabolic networks for the adaptation of *Pseudomonasaeruginosa* biofilms to urinary tract-like conditions. PLoS ONE 8(8) (2013) e71845. https://doi.org/10.1371/journal.pone.0071845 10.1371/journal.pone.0071845 23967252 PMC3742457

[27] Otto M.. Staphylococcal infections: mechanisms of biofilm maturation and detachment as critical determinants of pathogenicity. Annual Review in Medicine 64 (2013) 175-188. https://doi.org/10.1146/annurev-med-042711-140023 10.1146/annurev-med-042711-140023 22906361

[28] Wang Y.. Liposome as a delivery system for the treatment of biofilm-mediated infections. Journal of Applied Microbiology 131 (2021) 2626-2639. https://doi.org/10.1111/jam.15053 10.1111/jam.15053 33650748

[29] Lu T.K. Collins J.J.. Dispersing biofilms with engineered enzymatic bacteriophage. Proceedings of the National Academy of Sciences 104 (2007) 11197-202. https://doi.org/10.1073/pnas.0704624104 10.1073/pnas.0704624104 PMC1899193 17592147

[30] Nazir R. Zaffar M.R. Amin I.. Bacterial biofilms: the remarkable heterogeneous biological communities and nitrogen fixing microorganisms in lakes. Freshwater Microbiology 1 (2019) 307-340. https://doi.org/10.1016/B978-0-12-817495-1.00008-6 10.1016/B978-0-12-817495-1.00008-6

[31] Wood T.K. Knabel S.J. Kwan B.W.. Bacterial persister cell formation and dormancy. Applied Environmental Microbiology 79(79) (2013) 7116-7121. https://doi.org/10.1128/FAEM.02636-13 10.1128/FAEM.02636-13 24038684 PMC3837759

[32] Ramanathan S. Arunachalam K. Chandran S. Selvaraj R. Shunmugiah K. Arumugam V.. Biofilm inhibitory efficiency of phytol in combination with cefotaxime against nosocomial pathogen *Acinetobacter baumannii*. Journal of Applied Microbiology 125 (2018) 56-71. https://doi.org/ doi: 10.1111/jam.13741 10.1111/jam.13741 29473983

[33] Batoni G. Maisetta G. Esin S.. Antimicrobial peptides and their interaction with biofilms of medically relevant bacteria. Biochimical and Biophysic Acta (BBA) Biomembranes 1858 (2016) 1044-1060. https://doi.org/10.1016/i.bbamem.2015.10.013 10.1016/i.bbamem.2015.10.013 26525663

[34] Waldrop R. McLaren A. Calara F. McLemore R.. Biofilm growth has a threshold response to glucose in vitro. Clinical and Orthopedic Related Research 475(475) (2014) 3305-3310. https://doi.org/10.1007/s11999-014-3538-5 10.1007/s11999-014-3538-5 PMC4182383 24599648

[35] Purevdorj B. Costerton J.W. Stoodley P.. Influence of hydrodynamics and cell signaling on the structure and behavior of Pseudomonas aeruginosa biofilms. Applied Environmental Microbiology 68(68) (2002) 268-307. https://doi.org/10.1128/aem.68.9.4457-4464.2002 10.1128/aem.68.9.4457-4464.2002 PMC124093 12200300

[36] Munita J.M. Arias C.A.. Mechanisms of antibiotic resistance. Microbiology and Spectroscopy 4 (2016) VMBF-0016-2015. https://doi.org/10.1128/microbiolspec.VMBF-0016-2015 10.1128/microbiolspec.VMBF-0016-2015 PMC4888801 27227291

[37] Gupta T.T. Karki S.B. Fournier R. Ayan H.. Mathematical modelling of the effects of plasma treatment on the diffusivity of biofilm. Applied Sciences 8(8) (2018) 1729. https://doi.org/10.3390/app8101729 10.3390/app8101729

[38] Rather M.A. Gupta K. Mandal M.. Microbial biofilm: formation, architecture, antibiotic resistance, and control strategies. Brazilian Journal of Microbiology 52 (2021) 1701-1718. https://doi.org/10.1007/s42770-021-00624-x 10.1007/s42770-021-00624-x 34558029 PMC8578483

[39] Potera C.. Antibiotic resistance: biofilm dispersing agent rejuvenates older antibiotics. EnvironmentalHealth Perspective 118 (2010) 288-291. https://doi.org/10.1289/ehp.118-a288 10.1289/ehp.118-a288

[40] Smith A.W.. Biofilms and antibiotic therapy: is there a role for combating bacterial resistance by the use of novel drug delivery systems? Advances in Drug Delivery Review 57 (2005) 1539-1550. https://doi.org/10.1016/j.addr.2005.04.007 10.1016/j.addr.2005.04.007 15950314

[41] Hoiby N. Ciofu O. Johansen H.K. Song Z.J.. The clinical impact of bacterial biofilms. International Journal of Oral Sciences 3 (2011) 55-65.https://doi.org/10.4248/ijos11026 10.4248/ijos11026 PMC3469878 21485309

[42] Wolfmeier H. Pletzer D. Mansour S.C. Hancock R.E.. New perspectives in biofilm eradication. ACS Infectious Diseases 4 (2018) 93-106. https://doi.org/10.1021/acsinfecdis.7b00170 10.1021/acsinfecdis.7b00170 29280609

[43] Liu Y. Busscher H.J. Zhao B. Zhang Z. van der mei H.C. Ren Y. Busscher H.J.. Perspectives on and need to develop new infection control methods. In Racing for the Surface: Pathogenesis of Implant Infection and Advanced Antimicrobial Strategies,. Li B. Moriarty T.F. Webster T. Xing M., Eds., Springer Nature, Cham, Switzerland, 2019 p. 95. https://doi.org/10.1007/978-3-030-34475-7_5 10.1007/978-3-030-34475-7_5

[44] Neville N. Jia Z.. Approaches to the structure based design of antivirulence drugs: therapeutics for the post-antibiotic era. Molecules 24 (2019) 378. https://doi.org/10.3390/molecules24030378 10.3390/molecules24030378 30678155 PMC6384752

[45] Ramos M.D.A.S. Da Silva P.B. Sposito L. De Toledo L.G. Bonifacio B.V. Rodero C.F. Fernanda C. Karen D. Marlus C. Maria B Tais. Nanotechnology-based drug delivery systems for control of microbial biofilms. International Journal of Nanomedicine 13 (2018) 1179. https://doi.org/10.2147/ijn.s146195 10.2147/ijn.s146195 29520143 PMC5834171

[46] Podnecky N.L. Rhodes K.A. Schweizer H.P.. Efflux pump-mediated drug resistance in Burkholderia. Frontiers in Microbiology 6 (2015) 305. https://doi.org/10.3389/fmicb.2015.00305 10.3389/fmicb.2015.00305 25926825 PMC4396416

[47] Schlisselberg D.B. Kler E. Kisluk G. Shachar D. Yaron S.. Biofilm formation ability of Salmonella enterica serovar Typhimurium acrAB mutants. International Journal of Antimicrobial Agents 46 (2015) 456-459. https://doi.org/10.1016/j.ijantimicag.2015.06.011 10.1016/j.ijantimicag.2015.06.011 26260191

[48] Van Acker H. Van Dijck P. Coenye T.. Molecular mechanisms of antimicrobial tolerance and resistance in bacterial and fungal biofilms. Trends in Microbiology 22 (2014) 326-33. https://doi.org/10.1016/j.tim.2014.02.001 10.1016/j.tim.2014.02.001 24598086

[49] Qvortrup K. Hultqvist L.D. Nilsson M. Jakobsen T.H. Jansen C.U. Uhd J. Andersen J. Bo J. Thomas N.E. Micheal G. Tim T.. Small molecule anti-biofilm agents developed on the basis of mechanistic understanding of biofilm formation. Frontiers Chemistry 7 (2019)742. https://doi.org/10.3389/fchem.2019.00742 10.3389/fchem.2019.00742 PMC6838868 31737611

[50] Meng Y. Hou X. Lei J. Chen M. Cong S. Zhang Y. Ding W. Li G. Li X.. Multi-functional liposomes enhancing target and antibacterial immunity for antimicrobial and anti-biofilm against methicillin-resistant *Staphylococcus aureus*. Pharmaceutical Research 33 (2016) 763-775. https://doi.org/10.1007/s11095-015-1825-9 10.1007/s11095-015-1825-9 26666773

[51] Singh S. Singh S.K. Chowdgury I. Singh R.. Understanding the mechanism of bacterial biofilms resistance to antimicrobial agents. Open Microbiology Journal 11(2017) 53. https://doi.org/10.2174/1874285801711010053 10.2174/1874285801711010053 28553416 PMC5427689

[52] Stewart P.S. Franklin M.J.. Physiological heterogeneity in biofilms. Nature Review in Microbiology 6 (2008) 199-210. https://doi.org/10.1038/nrmicro1838 10.1038/nrmicro1838 18264116

[53] Forier K. Raemdonck K. De Smedt S.C. Demeester J. Coenye T. Braeckman K.. Lipid and polymer nanoparticles for drug delivery to bacterial biofilms. Journal of Control Release 190 (2014) 607-623. https://doi.org/10.1016/j.jconrel.2014.03.055 10.1016/j.jconrel.2014.03.055 24794896

[54] Albayaty Y.N. Thomas N. Hasan S. Prestidge C.A.. Penetration of topically used antimicrobials through *Staphylococcus aureus* biofilms: a comparative study using different models. Journal of Drug Delivery Science and Technology 48 (2018) 429-436. https://doi.org/10.1016/j.jddst.2018.10.015 10.1016/j.jddst.2018.10.015

[55] Kong X. Liu Y. Huang X. Huang S. Gao F. Rong P. Zhang S. Zhang K. Zeng W.. Cancer therapy based on smart drug delivery with advanced nanoparticles. Anticancer Agents Medicine and Chemistry 19 (2019) 720-730. https://doi.org/10.2174/1871520619666190212124944 10.2174/1871520619666190212124944 30747081

[56] Liu X. Xiang J. Zhu D. Jiang L. Zhou Z. Tang J. Liu X. Huang Y. Shen Y.. Fusogenic reactive oxygen species triggered charge-reversal vector for effective gene delivery. Advances in Material 28 (2016) 1743-1752. https://doi.org/10.1002/adma.201504288 10.1002/adma.201504288 26663349

[57] Lopes N.A. Brandelli A.. Nanostructures for delivery of natural antimicrobials in food. Critical Review in Food Sciences and Nutrition 58 (2018) 2201-2212. https://doi.org/10.1080/10408398.2017.1308915 10.1080/10408398.2017.1308915 28394691

[58] Kumar P. Shenoi R.A. Lia B.F. Nguyen M. Kizhakkedathu J.N. Straus S.K.. Conjugation of aurein 2.2 to HPG yields an antimicrobial with better properties. Biomacromolecule 16 (2015) 913-923. https://doi.org/10.1021/bm5018244 10.1021/bm5018244 25664972

[59] Zheng Y. Tai W.. Insight into the siRNA transmembrane delivery—from cholesterol conjugating to tagging. Wiley Interdiscipline Review in Nanomedicine and Nanobiotechnology 12 (2020) e1606. https://doi.org/10.1002/wnan.1606 10.1002/wnan.1606 31788983

[60] Bibi S. Lattmann E. Mohammed A.R. Perrie Y.. Trigger release liposome systems: local and remote controlled delivery? Journal of Microencapsule 29 (2012) 262-276. https://doi.org/10.3109/02652048.2011.646330 10.3109/02652048.2011.646330 22208705

[61] Mittal P. Saharan A. Verma R. Altalbawy F. Alfaidi M. Batiha G. Akter W. Gautam R.K. Uddin S. Rahman S.. Dendrimers: A New Race of Pharmaceutical Nanocarriers. BioMed Research International 2021 (2021) 8844030. https://doi.org/10.1155/2021/8844030 10.1155/2021/8844030 33644232 PMC7902124

[62] Kesharwani P. Banerjee S. Gupta U. Amin M.C.I.M. Padhye S. Sarkar F.H. Fazlul H. Arun I.K.. PAMAM dendrimers as promising nanocarriers for RNAi therapeutics. Materials Today 18 (2015) 565-572. https://doi.org/10.1016/j.mattod.2015.06.003 10.1016/j.mattod.2015.06.003

[63] Namazi H. Hamrahloo Y.T.. Novel pH sensitive nanocarrier agents based on citric acid dendrimers containing conjugated b-cyclodextrins. Advances in Pharmaceutical Bulletin 1(1) (2011) 40-47. https://doi.org/10.5681/apb.2011.006 10.5681/apb.2011.006 PMC3850000 24312755

[64] Surekha B. Kommana N.S. Dubey S.K. Kumar A.V.P. Shukla P. Kesharwani P.. PAMAM dendrimer as a talented multifunctional biomimetic nanocarrier for cancer diagnosis and therapy. Colloids Surface B 204 (2021) 111837. https://doi.org/10.1016/j.colsurfb.2021.111837 10.1016/j.colsurfb.2021.111837 33992888

[65] Choudhury H. Sisinthy S.P. Gorain B. Kesharwani P.. History and introduction of dendrimers, dendrimer-based nanotherapeutics, Kesharwani P., Ed., Elsevier, Kuala Lumpur, Malaysia, 2021. p. 1-14. https://doi.org/10.1016/b978-0-12-821250-9.00014-7 10.1016/b978-0-12-821250-9.00014-7

[66] Gawande V. Choudhury V. Kesharwani P.. Dendrimer nomenclature and synthesis methods, dendrimer-Based Nanotherapeutics, Kesharwani P., Ed., Elsevier, Pune, India, 2021, p. 75-94. https://doi.org/10.1016/b978-0-12-821250-9.00009-3 10.1016/b978-0-12-821250-9.00009-3

[67] Jian K. Jain N. Kesharwani P. Kesharwani P.. Types of dendrimers, dendrimer-based nanotherapeutics, Kesharwani P., Ed., Elsevier, Raebareli, India, 2021, p. 95-123. https://doi.org/10.1016/b978-0-12-821250-9.00007-x 10.1016/b978-0-12-821250-9.00007-x

[68] Luong D. Kesharwani P. Deshmukh R. Amin M. Gupta U. Greish K. Arun I.K.. PEGylated PAMAM dendrimers: enhancing efficacy and mitigating toxicity for effective anticancer drug and gene delivery. Acta Biomaterial 43 (2016) 14-29. https://doi.org/10.1016/j.actbio.2016.07.015 10.1016/j.actbio.2016.07.015 27422195

[69] Singh B. Saini S. Lohan S. Beg S., Systematic development of nanocarriers employing quality by design paradigms, in Nanotechnology-based approaches for targeting and delivery of drugs and genes, Kesharwani P. Mishra V. Mohd Amin M.C.I. Iyer A., Eds.,Academic Press, 2017. p. 110-148. https://doi.org/10.1016/b978-0-12-809717-5.00003-8 10.1016/b978-0-12-809717-5.00003-8

[70] Singh S. Singh G. Sehrawat S. Rawat P. Molugulu N. Singh V. Ahmed F.J. Kesharwani P.. Future considerations of dendrimers, Dendrimer-based nanotherapeutics, Kesharwani P., Ed., Elsevier, Punjab, India, 2021, p. 449-458. https://doi.org/10.1016/b978-0-12-821250-9.00005-6 10.1016/b978-0-12-821250-9.00005-6

[71] Bashiz N.J. Asefnejad A. Saadatabad A.R.. A polycaprolactone/cellulose acetate/polycaprolactone scaffolds: Study the absorption, kinetics and controlled release of anticancer drugs. Iranian Journal of Chemistry and Chemical Engineering (2023). https://doi.org/10.30492/ijcce.2023.2009095.6173 10.30492/ijcce.2023.2009095.6173

[72] Sherje A.P. Jadhav M. Dravyakar B.R. Darshana K.. Dendrimers: a versatile nanocarrier for drug delivery and targeting. International Journal of Pharmacy 548 (2018) 707-720. https://doi.org/10.1016/j.ijpharm.2018.07.030 10.1016/j.ijpharm.2018.07.030 30012508

[73] Pooresmaeil M. Namazi H.. Advances in development of the dendrimers having natural saccharides in their structure for efficient and controlled drug delivery applications. European Polymer Journal 148 (2021) 110356. https://doi.org/10.1016/j.eurpolymj.2021.110356 10.1016/j.eurpolymj.2021.110356

[74] Gorain B. Pandey M. Choudhury H. Jain G.K. Kesharwani P.. Dendrimer for solubility enhancement, dendrimer-based nanotherapeutics, Kesharwani P., Ed., D Elsevier, Selangor, Malaysia, 2021, p. 273-83. https://doi.org/10.1016/b978-0-12-821250-9.00025-1 10.1016/b978-0-12-821250-9.00025-1

[75] Patel V. Rajani C. Paul D. Borisa P. Rajpoot K. Youngren-Ortiz R.S. Tekade R.K.. Dendrimers as novel drug-delivery system and its applications, Drug delivery systems, Takade R. K., Ed., .Elsevier, Ahmedabad, India, 2019, p. 333-392. https://doi.org/10.1016/b978-0-12-814487-9.00008-9 10.1016/b978-0-12-814487-9.00008-9

[76] Hosseyni R. Pooresmaeil M. Namazi H H.. Star-shaped polylactic acid-based triazine dendrimers: the catalyst type and time factors influence on polylactic acid molecular weight. Polymer 29(29) (2020) 423-432. https://doi.org/10.1007/s13726-020-00807-7 10.1007/s13726-020-00807-7

[77] Soni N. Jain U. Gupta U. Jain N.. Controlled delivery of Gemcitabine Hydrochloride using mannosylated poly (propyleneimine) dendrimers. Journal of Nanoparticles Research 17(17) (2015) 458. https://doi.org/10.1007/s11051-015-3265-1 10.1007/s11051-015-3265-1

[78] Karthikeyan R. Koushik O.S. Kumar P.V.. Dendrimeric architecture for effective antimicrobial therapy, dendrimers for drug delivery, dendrimers for controlled release drug delivery, Apple Academic Press, Palm Bay, USA, 2019,p. 375-405. https://www.researchgate.net/publication/331033521

[79] Authimoolam S. Dziubla T.. Biopolymeric mucin and synthetic polymer analogs: Their structure, function and role in biomedical applications. Polymers 8 (2016) 71. https://doi.org/10.3390/polym8030071 10.3390/polym8030071 30979166 PMC6432556

[80] Cheng Y. Qu H. Ma M. Xu Z. Xu P. Fang Y. Xu T.. Polyamidoamine (PAMAM) dendrimers as biocompatible carriers of quinolone antimicrobials: An in vitro study. European Journal of Medical Chemistry 42 (2007) 1032-1038. https://doi.org/10.1016/j.ejmech.2006.12.035 10.1016/j.ejmech.2006.12.035 17336426

[81] Kuwahara K. Kitazawa T. Kitagaki H. Tsukamoto T. Kikuchi M.. Nadifloxacin, an antiacne quinolone antimicrobial, inhibits the production of proinflammatory cytokines by human peripheral blood mononuclear cells and normal human keratinocytes. Journal of Dermatological Sciences 38 (2005) 47-55. https://doi.org/10.1016/j.jdermsci.2005.01.002 10.1016/j.jdermsci.2005.01.002 15795123

[82] Svenningsen S.W. Frederiksen R.F. Counil C. Ficker M. Leisner J.J. Christensen J.B.. Synthesis and Antimicrobial Properties of a Ciprofloxacin and PAMAM-dendrimer Conjugate. Molecules 25 (2020) 1389. https://doi.org/10.3390/molecules25061389 10.3390/molecules25061389 32197523 PMC7146445

[83] Bugno J. Hsu H.J. Hong S.. Tweaking dendrimers and dendritic nanoparticles for controlled nano-bio interactions: Potential nanocarriers for improved cancer targeting. Journal of Drug Targeting 23(7-8) (2015) 642-650. https://doi.org/10.3109/1061186X.2015.1052077 10.3109/1061186X.2015.1052077 26453160 PMC4980844

[84] Nanjwade B.K. Bechra H.M. Derkar G.K. Manvi F. Nanjwade V.K.. Dendrimers: emerging polymers for drug-delivery systems. European Journal of Pharmaceutical Sciences 38(38) (2009) 185-196. https://doi.org/10.1016/j.ejps.2009.07.008 10.1016/j.ejps.2009.07.008 19646528

[85] Jain K... 7 - Dendrimers: Smart nanoengineered polymers for bioinspired applications in drug delivery, Biopolymer-based composites, Jana S. Maiti S. Jana S. Eds., Woodhead Publishing, Raebareli, India,2017 p. 169-220. https://doi.org/10.1016/B978-0-08-101914-6.00007-7 10.1016/B978-0-08-101914-6.00007-7

[86] Kesrevani R.K. Sharma A.K.. Nan*oarchitectured biomaterials: present status and future prospects in drug deliver*y, in Nanoarchitectonics for smart delivery and drug targeting, Grumezescu A.M. Ed., Elsevier Inc, Amsterdam, Netherlands, 2016 p. 35-66. ISBN: 9780323477222

[87] Santos A. Veiga F. Figueiras A.. Dendrimers as pharmaceutical excipients: Synthesis, properties, toxicity and biomedical applications. Materials 13(1) (2019) 65. https://doi.org/10.3390/ma13010065 10.3390/ma13010065 31877717 PMC6981751

[88] Lyu Z. Ding L. Huang A.Y. Kao C. Peng L.. Poly (amidoamine) dendrimers: covalent and supramolecular synthesis. Materials Today Chemistry 13 (2019) 34-48. https://doi.org/10.1016/j.mtchem.2019.04.004 10.1016/j.mtchem.2019.04.004

[89] Tomalia D.A. Baker H Dewald J. Hall M. Kallos G. Martin S Ryder J. Smith P.. A New class of polymers: starburst-dendritic macromolecules. Polymer Journal 17 (1985) 117-132. https://doi.org/10.1295/polymj.17.117 10.1295/polymj.17.117

[90] Abbasi E. Aval S.F. Akbarzadeh A. Milani M. Nasrabadi H. Joo S.W.. Dendrimers: Synthesis, applications, and properties. Nanoscale Research Letters 9(9) (2014)247. https://doi.org/10.1186/1556-276X-9-247 10.1186/1556-276X-9-247 24994950 PMC4074873

[91] Caminade A.M. Turrin C.O. Laurent R. Maraval A. Majoral J.P.. Synthetic pathways towards phosphorus dendrimers and dendritic architectures. Current Organic Chemistry 10 (2006) 2333-2355. https://doi.org/10.2174/138527206778992680 10.2174/138527206778992680

[92] Hawker C.J. Fréchet J.M.J. Preparation of polymers with controlled molecular architecture. A new convergent approach to dendritic macromolecules. Journal of the American Chemical Society 112 (1990) 7638-7646. https://doi.org/10.1021/ja00177a027 10.1021/ja00177a027

[93] Tripathy S. Baro L. Das M.K.. Dendrimer chemistry and host-guest interactions for drug targeting. International Journal of Pharmaceutical Science and Research 5(1) (2014) 16-25. http://dx.doi.org/10.13040/IJPSR.0975-8232.5(1).16-25 10.13040/IJPSR.0975-8232.5(1).16-25

[94] Gillies E.R. Fréchet J.M.J.. Designing macromolecules for therapeutic applications: polyester dendrimer poly(ethylene oxide) “bow-tie” hybrids with tunable molecular weight and architecture. Journal of the American Chemical Society 124(124) (2002) 14137-14146. https://doi.org/10.1021/ja028100n 10.1021/ja028100n 12440912

[95] Lee J.W. Kim H.H. Kim H.J. Han S.C. Kim J.H. Shin W.S. Jin S.. Synthesis of symmetrical and unsymmetrical PAMAM dendrimers by fusion between azide- and alkyne-functionalized PAMAM dendrons. Bioconjugate Chemistry 18(18) (2007) 579-584. https://doi.org/10.1021/bc060256f 10.1021/bc060256f 17335177

[96] Augustus E.N. Allen E.T. Nimibofa A. Donbebe W.. A review of synthesis, characterization and applications of functionalized dendrimers. American Journal of Polymer Science 7(7) (2017) 8-14. https://doi.org/10.5923/j.ajps.20170701.02 10.5923/j.ajps.20170701.02

[97] Donnio B. Buathong S. Bury I. Guillon D.. Liquid crystalline dendrimers. Chemical Society Reviews 36(9) (2007) 495-513. https://doi.org/10.1039/b605531c 10.1039/b605531c 17660881

[98] Gurunathan S. Kang M.H. Qasim M. Kim J.H.. Nanoparticle-mediated combination therapy: two-in-one approach for cancer. International Journal of Molecular Sciences 19(19) (2018) 3264. https://doi.org/10.3390/ijms19103264 10.3390/ijms19103264 30347840 PMC6214025

[99] Jain A. Dubey S. Kaushik A. Tyagi K.. Dendrimer: a complete drug carrier. International Journal of Pharmaceutical Science Research 1(1) (2010) 38-52. http://dx.doi.org/10.13040/IJPSR.0975-8232 10.13040/IJPSR.0975-8232

[100] Joshi V.G. Dighe V.D. Thakuria D. Malik Y.S. Kumar S.. Multiple antigenic peptide (MAP): a synthetic peptide dendrimer for diagnostic, antiviral and vaccine strategies for emerging and re-emerging viral diseases. Indian Journal of Virology 24(24) (2013) 312-320. https://doi.org/10.1007/s13337-013-0162-z 10.1007/s13337-013-0162-z 24426293 PMC3832690

[101] Kalomiraki M. Thermos K. Chaniotakis N.A.. Dendrimers as tunable vectors of drug delivery systems and biomedical and ocular applications. International Journal of Nanomedicine 11 (2015) 1-12. https://doi.org/10.2147/ijn.s93069 10.2147/ijn.s93069 26730187 PMC4694674

[102] Longley D.B. Harkin D.P. Johnston P.G.. 5-Fluorouracil: Mechanisms of action and clinical strategies. Nature Reviews Cancer 3(3) (2003) 330-338. https://doi.org/10.1038/nrc1074 10.1038/nrc1074 12724731

[103] Ramalingam K. Frohlich N.C. Lee A.C.. Effect of nanoemulsion on dental unit waterline biofilm. Journal Dental 28 (2013) 333-336. https://doi.org/10.1016/j.jds.2013.02.035 10.1016/j.jds.2013.02.035

[104] Janiszewska J. Swieton J. Lipkowski A.W. Urbanczyk-Lipkowska Z.. Low molecular mass peptide dendrimers that express antimicrobial properties. Bioorganism Medicine and Chemistry Letters 13 (2003) 3711-3713. https://doi.org/10.1016/j.bmcl.2003.08.009 10.1016/j.bmcl.2003.08.009 14552763

[105] Johansson E.M.W. Crusz S.A. Kolomiets E. Buts L. Kadam R.U. Cacciarini M. Bartels K. Diggle S.P. Camara M. Williams P. Loris R. Nativi C. Rosenau F. Jaeger K. Darbre T. Reymond J.. Inhibition and dispersion of *Pseudomonas aeruginosa* biofilms by glycopeptide dendrimers targeting the fucose specific lectin LecB. Chemistry and Biology 15 (2008) 1249-1257. https://doi.org/10.1016/j.chembiol.2008.10.009 10.1016/j.chembiol.2008.10.009 19101469

[106] Dhumal D. Maron B. Malach M. Ding L. Marson D. Laurini E. Tintaru A. Ralahy B. Giorgio S. Pricl S. Hayouka Z. Peng L.. Dynamic self-assembling supramolecular dendrimer nanosystems as potent antibacterial candidates against drug-resistant bacteria and biofilms. Nanoscale 14 (2022) 9286. https://doi.org/10.1039/d2nr02305a 10.1039/d2nr02305a 35649277

[107] Llamazares C. Sanz Del Olmo N. Ortega P. Gomez R. Soliveri J. de la Mata F.J. Garcia-Gallego S. Copa-Patino J.L.. Antibacterial effect of carbosilanemetallo dendrimers in planktonic cells of gram-positive and gram-negative bacteria and Staphylococcus aureus biofilm. Biomolecules 9(9) (2019) 405. https://doi.org/10.3390/biom9090405 10.3390/biom9090405 31450779 PMC6769849

[108] Han X. Liu Y. Ma Y. Zhang M. He Z. Siriwardena T.N. Xu H Bai Y. Zhang X. Reymond J.L. Qiao M.. Peptide dendrimers G3KL and TNS18 inhibit *Pseudomonas aeruginosa* biofilms. Applied Microbiology Biotechnology 103 (2019) 5821-5830. https://doi.org/10.1007/s00253-019-09801-3 10.1007/s00253-019-09801-3 31101943

[109] Gide M. Nimmagadda A. Su M. Wang M. Teng P. Li C. Gao R. Xu H. Li Q. Cai J.. Nano-Sized Lipidated dendrimers as potent and broad-spectrum antibacterial agents. Macromolocular and Rapid Communication 39 (2018) 1800622. https://doi.org/10.1002/marc.201800622 10.1002/marc.201800622 30408252

[110] Rozenbaum R.T Andrén O.G.C. van der Mei H.C. Woudstra W. Busscher H.J. Malkoch M. Sharma P.K.. Penetration and accumulation of dendrons with different peripheral composition in *Pseudomonas aeruginosa* biofilms. Nano Letters 19 (2019) 4327-4333. https://doi.org/10.1021/acs.nanolett.9b00838 10.1021/acs.nanolett.9b00838 31142116 PMC6628176

[111] Andreozzi E. Barbieri F. Ottavian M.F. Giorgi L. Bruscolini F. Manti A. Battisetelli M. Sabatini L. Pianetti A.. Dendrimers and Polyamino-Phenolic Ligands: Activity of New Molecules Against Legionella pneumophila Biofilms. Frontiers in Microbiology 7 (2016) 289. https://doi.org/10.3389/fmicb.2016.00289 10.3389/fmicb.2016.00289 27014213 PMC4783402

[112] Bergmann M. Michaud G. Visini R. Jin X. Gillon E. Stocker A. Imberty A. Darbre T. Reymond J.L.. Multivalency effects on *Pseudomonas aeruginosa* biofilm inhibition and dispersal by glycopeptide dendrimers targeting lectin LecA. Organic and Biomolecular Chemistry 14 (2016) 138. https://doi.org/10.1039/c5ob01682g 10.1039/c5ob01682g 26416170

[113] Michaud G. Visini R. Bergmann M. Salerno G. Bosco R. Gillon E. Richichi B. Nativi C. Imberty A. Stocker A. Darbre T. Reymond J.L.. Overcoming antibiotic resistance in *Pseudomonas aeruginosa* biofilms using glycopeptide dendrimers. Chemical Science 7 (2016) 166. https://doi.org/10.1039/c5sc03635f 10.1039/c5sc03635f 29896342 PMC5953009

[114] Emma M. Johansson A. Kolomiets E. But L. Kadam R.U. Cacciarini M. Bartels K. Diggle S.P. Camara M. Williams P. Loris R. Nativi C. Rosenau F. Jaeger K. Darbre T. Reymond J.L.. Inhibition and Dispersion of *Pseudomonas aeruginosa* Biofilms by Glycopeptide Dendrimers Targeting the Fucose-Specific Lectin LecB. Chemistry & Biology 15 (2008) 1249-1257. https://doi.org/10.1016/j.chembiol.2008.10.009 10.1016/j.chembiol.2008.10.009 19101469

[115] Huh A.J. Kwon Y.J.. Nanoantibiotic: a new paradigm for treating infectious diseases using nanomaterials in the antibiotics-resistant era. Journal of Control Release 156 (2011) 128-145. https://doi.org/10.1016/j.jconrel.2011.07.002 10.1016/j.jconrel.2011.07.002 21763369

[116] Singh U. Dar M.M. Hashmi A.A.. Dendrimers: synthetic strategies, properties and applications. Oriental Journal of Chemistry 30(30) (2014) 911-922. https://doi.org/10.13005/ojc/300301 10.13005/ojc/300301

[117] Mignani S. Shi X. Cena V. Rodrigues J. Tomas H. Majoral J.. Jean-Pierre MajoralEngineered non-invasive functionalized dendrimer/ dendron-entrapped/complexed gold nanoparticles as a novel class of theranostic (radio)pharmaceuticals in cancer therapy. Journal of Control Release 332 (2021) 346-366. https://doi.org/10.1016/j.jconrel.2021.03.003 10.1016/j.jconrel.2021.03.003 33675878

[118] Kim Y. Park E.J. Na D.H.. Recent progress in dendrimer-based nanomedicine development. Archive Pharmacy 41 (2018) 571-582. https://doi: 10.1007/s12272-018-1008-4 10.1007/s12272-018-1008-4 29450862

[119] Janaszewska A. Lazniewska J. Trzepinski P. Marcinkowska M. Klajnert-Maculewicz B.. Cytotoxicity of dendrimers. Biomolecules 9 (2019) 330. https://doi.org/10.3390/biom9080330 10.3390/biom9080330 31374911 PMC6723213

[120] Falanga A. Del Genio V. Galdiero S.. Peptides and Dentdrimers: How to combat viral and bacterial infections. Pharmaceutics 13 (2021) 101. https://doi.org/10.3390/pharmaceutics13010101 10.3390/pharmaceutics13010101 33466852 PMC7830367

[121] Sharifi S. ZununiVahed S. Jahangiri A.. Dendrimers as drug delivery systems; the benefits and challenges. Journal of Advance in Chemistry and Pharmaceutical Material 2(2) (2019) 119-123. https://api.semanticscholar.org/CorpusID:210908029

[122] Kannan R.M. Nance E. Kannan S. Tomalia D.A.. Emerging concepts in dendrimer-based nanomedicine: from design principles to clinical applications. Journal of Intermediary Medicine 276 (2014) 579-617. https://doi.org/10.1111/joim.12280 10.1111/joim.12280 24995512

[123] Liu S. Cai X. Xue W. Ma D. Zhang W.. Chitosan derivatives co-delivering nitric oxide and methicillin for the effective therapy to the methicillin-resistant *S. aureus* infection. Carbohydrate and Polymer 234 (2020) 115928. https://doi.org/10.1016/j.carbpol.2020.115928 10.1016/j.carbpol.2020.115928 32070544

[124] Qi X. Qin Y. Fan X. Qin Y. Jiang Z. Wu Z.. Carboxymethyl Chitosan-Modified Polyamidoamine Dendrimer Enables Progressive Drug Targeting of Tumors via pH-Sensitive Charge Inversion. Journal of Biomedicine and Nanotechnology 12(12) (2016) 667-678. https://doi.org/10.1166/jbn.2016.2206 10.1166/jbn.2016.2206 27301193

[125] Yang H.. Targeted nanosystems: advances in targeted dendrimers for cancer therapy. Nanomedicine 12 (2016) 309-316. https://doi.org/10.1016/j.nano.2015.11.012 10.1016/j.nano.2015.11.012 26706410 PMC4789125

[126] Zhou L. Gan L. Li H. Yang X.. Studies on the interactions between DNA and PAMAM with fluorescent probe [Ru(phen)2d ppz]2+. Journal of Pharmaceutical Biomedicine 43 (2007) 330-334. https://doi.org/10.1016/j.jpba.2006.06.021 10.1016/j.jpba.2006.06.021 16872783

[127] de Araujo R.V. Santos S.S. Ferreira E.I. Giarolla J.. New advances in general biomedical applications of PAMAM dendrimers. Molecules 23 (2018) 2849. https://doi.org/10.3390/molecules23112849 10.3390/molecules23112849 30400134 PMC6278347

[128] Pryor J.B. Harper B.J. Harper S.L.. Comparative toxicological assessment of PAMAM and thiophosphoryl .B dendrimers using embryonic zebrafish. International Journal of Nanomedicine 9 (2014) 1947-1956. https://doi.org/10.2147/ijn.s60220 10.2147/ijn.s60220 24790436 PMC4000179

[129] VanKoten H.W. Dlakic W.M. Engel R. Cloninger M.J.. Synthesis and biological activity of highly cationic dendrimer antibiotics. Molecular Pharmacology 13 (2016) 3827-3834. https://doi.org/10.1021/acs.molpharmaceut.6b00628 10.1021/acs.molpharmaceut.6b00628 PMC9389851 27661609

[130] Worley B.V. Schilly K.M. Schoenfisch M.H.. Anti-Biofilm efficacy of dual-action nitric oxide-releasing alkyl chain modified poly(amidoamine) dendrimers. Molecular Pharmacology 12 (2015) 1573-1583. https://doi.org/10.1021/acs.molpharmaceut.5b00006 10.1021/acs.molpharmaceut.5b00006 25873449

[131] Fang F.C.. Perspectives series: host/pathogen interactions. Mechanisms of nitric oxide-related antimicrobial activity. Journal of Clinical Investigation 99 (1997) 2818-2825. https://doi.org/10.1172/jci119473 10.1172/jci119473 9185502 PMC508130

[132] Mehrizi T.Z. Ardestani M.S. Kafiabad S.A.. Review Study of dendrimer nanoparticles influences on stored platelet in order to treat patients (2001-2020). Current Nanoscience 17 (2021) 304-318. https://doi.org/10.2174/1566524021666210708154736 10.2174/1566524021666210708154736

[133] Han M.H. Chen J. Wang J. Chen S.L. Wang X.T.. Blood compatibility of polyamidoamine dendrimers and erythrocyte protection. Journal of Biomedical and Nanotechnology 6 (2010) 82-92. https://doi.org/10.1166/jbn.2010.1096 10.1166/jbn.2010.1096 20499836

[134] Duncan R. Izzo L.. L. Dendrimer biocompatibility and toxicity. Advance in Drug Delivery Review 57 (2005) 2215-2237.https://doi.org/10.1016/j.addr.2005.09.019 10.1016/j.addr.2005.09.019 16297497

[135] Dabkowska M. Ulańczyk Z. Łuczkowska K. Rogińska D. Sobuś A Wasilewska M. Olszewska M. Jakubowska K. Machalinski B.. The role of the electrokinetic charge of neurotrophis-based nanocarriers: Protein distribution, toxicity, and oxidative stress in in vitro setting. Journal of Nanobiotechnology 19 (2021) 258. https://doi.org/10.1186/s12951-021-00984-4 10.1186/s12951-021-00984-4 34454520 PMC8399784

[136] Thiagarajan G. Greish K. Ghandehari H.. Charge affects the oral toxicity of poly(amidoamine) dendrimers. European Journal of Pharmacy and Biopharmacy 84 (2013) 330-334. https://doi.org/10.1016/j.ejpb.2013.01.019 10.1016/j.ejpb.2013.01.019 PMC3860365 23419816

[137] Enciso A.E. Neun B. Rodriguez J. Ranjan A.P. Dobrovolskaia M.A. Simanek E.E.. Nanoparticle Effects on Human Platelets in Vitro: A Comparison between PAMAM and Triazine Dendrimers. Molecules 21 (2016) 428. https://doi.org/10.3390/molecules21040428 10.3390/molecules21040428 27043508 PMC6273833

[138] Dobrovolskaia M.A. Patri A.K. Simak J. Hall J.B Semberova J. De Paoli Lacerda S.H. Silvia H McNeil S.E.. Nanoparticle size and surface charge determine effects of PAMAM dendrimers on human platelets in vitro. Molecular Pharmaceutics 9 (2012) 382-393.https://doi.org/10.1021/mp200463e 10.1021/mp200463e 22026635 PMC3624701

[139] Hannon G. Lysaght J. Liptrott N.J. Prina-Mello A.. Immunotoxicity Considerations for Next Generation Cancer Nanomedicines. Advances in Science 6 (2019) 1900133. https://doi.org/10.1002/advs.201900133 10.1002/advs.201900133 PMC6774033 31592123

[140] Roberts J.C. Bhalgat M.K. Zera R.T.. Preliminary biological evaluation of polyamidoamine (PAMAM) Starburst dendrimers. Journal of Biomedical and Material Research 30 (1996) 53-65. https://doi.org/10.1002/(SICI)1097-4636(199601)30:1%3C53::AID-JBM8%3E3.0.CO;2-Q 10.1002/(SICI)1097-4636(199601)30:1<53::AID-JBM8>3.0.CO;2-Q 8788106

[141] Albertazzi L. Gherardini L. Brondi M. Sulis Sato S. Bifone A. Pizzorusso T. Ratto G.M. Bardi G.. In vivo distribution and toxicity of PAMAM dendrimers in the central nervous system depend on their surface chemistry. Molecular Pharmaceutics 10 (2013) 249-260. https://doi.org/10.1021/mp300391v 10.1021/mp300391v 23163881

[142] Ayub A. Wettig S.. An Overview of Nanotechnologies for Drug Delivery to the Brain. Pharmaceutics 14 (2022) 224. https://doi.org/10.3390/pharmaceutics14020224 10.3390/pharmaceutics14020224 35213957 PMC8875260

[143] Thiagarajan G. Sadekar S. Greish K. Ray A. Ghandehari H.. Evidence of oral translocation of anionic G6.5 dendrimers in mice. Molecular Pharmaceutics 10 (2013) 988-998. https://doi.org/10.1021/mp300436c 10.1021/mp300436c 23286733 PMC3715149

[144] Aillon K.L. Xie Y. El-Gendy N. Berkland C.J. Forrest M.L.. Effects of nanomaterial physicochemical properties on in vivo toxicity. Advance Drug Delivery Review 61 (2009) 457-466. https://doi.org/10.1016/j.addr.2009.03.010 10.1016/j.addr.2009.03.010 PMC2743376 19386275

[145] Sadekar S. Ghandehari H.. Transepithelial transport and toxicity of PAMAM dendrimers: Implications for oral drug delivery. Advance Drug Delivery Review 64 (2012) 571-588. https://doi.org/10.1016/j.addr.2011.09.010 10.1016/j.addr.2011.09.010 PMC3305851 21983078

[146] Li Y. Zeng X. Wang S. Sun Y. Wang Z. Fan J. Song P. Ju D.. Inhibition of autophagy protects against PAMAM dendrimers-induced hepatotoxicity. Nanotoxicology 9 (2015) 344-355. https://doi.org/10.3109/17435390.2014.930533 10.3109/17435390.2014.930533 24983897

[147] Yellepeddi V.K. Ghandehari H.. Poly (amido amine) dendrimers in oral delivery. Tissue Barriers 4 (2016) e1173773. https://doi.org/10.1080/21688370.2016.1173773 10.1080/21688370.2016.1173773 27358755 PMC4910834

[148] Hubbard D. Ghandehari H. Brayden D.J.. Transepithelial transport of PAMAM dendrimers across isolated rat jejunal mucosae in ussing chambers. Biomacromolecules 15 (2014) 2889-2895. https://doi.org/10.1021/bm5004465 10.1021/bm5004465 24992090 PMC4130240

[149] Qu F. Geng R. Liu Y. Zhu J.. Advanced nanocarrier-and microneedle-based transdermal drug delivery strategies for skin diseases treatment. Theranostics 12 (2022) 3372. https://doi.org/10.7150/thno.69999 10.7150/thno.69999 35547773 PMC9065205

[150] Gao Y. Wang J. Chai M. Li X. Deng Y. Jin Q. Ji J.. Size and charge adaptive clustered nanoparticles targeting the biofilm microenvironment for chronic lung infection management. ASC Nano 14 (2020) 5686-5699. https://doi.org/10.1021/acsnano.0c00269 10.1021/acsnano.0c00269 32320228

[151] Mohapatra A. Uthaman S. Park I.K.. Polyethylene glycol nanoparticles as promising tools for anticancer therapeutics, Polymeric nanoparticles as a promising tool for anticancer therapeutics, Kesharwani P. Paknikar K.M. Gajbhiye V., Eds., Elsevier, Gwangju, Republic of Korea, 2019 p. 205-231 .https://doi.org/10.1016/b978-0-12-816963-6.00010-8 10.1016/b978-0-12-816963-6.00010-8

[152] Ho M.N. Bach L.G. Nguyen D.H. Nguyen C.H. Nguyen C.K. Tran Q.N. Nguyen N.V. Hoang Thi T.T.. PEGylated PAMAM dendrimers loading oxaliplatin with prolonged release and high payload without burst effect. Biopolymers 110(110) (2019) e23272. https://doi.org/10.1002/bip.23272 10.1002/bip.23272 30897210

[153] Zhu S. Hong M. Zhang L. Tang G. Jiang Y. Pei Y.. PEGylated PAMAM dendrimerdoxorubicin conjugates: in vitro evaluation and in vivo tumor accumulation. Pharmaceutical Research 27 (2009) 162-174. https://doi.org/10.1007/s11095-009-9992-1 10.1007/s11095-009-9992-1 19862607

[154] Wang G. Zhu D. Zhou Z. Piao Y. Tang J. Shen Y.. A glutathione-specific and intracellularly labile polymeric nanocarrier for efficient and safe cancer gene delivery. ACS Applied Material Interface 12 (2020) 14825-14838. https://doi.org/10.1021/acsami.9b22394 10.1021/acsami.9b22394 32166948

[155] Parsian M. Mutlu P. Yalcin S. Tezcaner A. Gunduz U.. Half generations magnetic PAMAM dendrimers as an effective system for targeted gemitabine delivery. International Journal of Pharmacy 515 (2016) 104-113. https://doi.org/10.1016/j.ijpharm.2016.10.015 10.1016/j.ijpharm.2016.10.015 27725272

